# Comparative *In Vitro* and *In Silico* Analyses of Variants in Splicing Regions of *BRCA1* and *BRCA2* Genes and Characterization of Novel Pathogenic Mutations

**DOI:** 10.1371/journal.pone.0057173

**Published:** 2013-02-22

**Authors:** Mara Colombo, Giovanna De Vecchi, Laura Caleca, Claudia Foglia, Carla B. Ripamonti, Filomena Ficarazzi, Monica Barile, Liliana Varesco, Bernard Peissel, Siranoush Manoukian, Paolo Radice

**Affiliations:** 1 Unit of Molecular bases of genetic risk and genetic testing, Department of Preventive and Predictive Medicine, Fondazione IRCCS Istituto Nazionale dei Tumori, Milano, Italy; 2 Cogentech, Consortium for Genomic Technologies, IFOM-IEO Campus, Milano, Italy; 3 IFOM, Fondazione Istituto FIRC di Oncologia Molecolare, Milano, Italy; 4 Division of Cancer Prevention and Genetics, Istituto Europeo di Oncologia, Milano, Italy; 5 Unit of Hereditary Cancer, Department of Epidemiology, Prevention and Special Functions, IRCCS San Martino IST- Istituto Nazionale per la Ricerca sul Cancro, Genova, Italy; 6 Unit of Medical Genetics, Department of Preventive and Predictive Medicine, Fondazione IRCCS Istituto Nazionale Tumori, Milano, Italy; University of Illinois at Chicago, United States of America

## Abstract

Several unclassified variants (UVs) have been identified in splicing regions of disease-associated genes and their characterization as pathogenic mutations or benign polymorphisms is crucial for the understanding of their role in disease development. In this study, 24 UVs located at *BRCA1* and *BRCA2* splice sites were characterized by transcripts analysis. These results were used to evaluate the ability of nine bioinformatics programs in predicting genetic variants causing aberrant splicing (spliceogenic variants) and the nature of aberrant transcripts. Eleven variants in *BRCA1* and 8 in *BRCA2*, including 8 not previously characterized at transcript level, were ascertained to affect mRNA splicing. Of these, 16 led to the synthesis of aberrant transcripts containing premature termination codons (PTCs), 2 to the up-regulation of naturally occurring alternative transcripts containing PTCs, and one to an in-frame deletion within the region coding for the DNA binding domain of BRCA2, causing the loss of the ability to bind the partner protein DSS1 and ssDNA. For each computational program, we evaluated the rate of non-informative analyses, i.e. those that did not recognize the natural splice sites in the wild-type sequence, and the rate of false positive predictions, i.e., variants incorrectly classified as spliceogenic, as a measure of their specificity, under conditions setting sensitivity of predictions to 100%. The programs that performed better were Human Splicing Finder and Automated Splice Site Analyses, both exhibiting 100% informativeness and specificity. For 10 mutations the activation of cryptic splice sites was observed, but we were unable to derive simple criteria to select, among the different cryptic sites predicted by the bioinformatics analyses, those actually used. Consistent with previous reports, our study provides evidences that *in silico* tools can be used for selecting splice site variants for *in vitro* analyses. However, the latter remain mandatory for the characterization of the nature of aberrant transcripts.

## Introduction

It is estimated that approximately 5% to 10% of all breast cancers occur in women with a positive family history, and that approximately 15% to 25% of familial aggregations are due to deleterious germline mutations affecting either the *BRCA1* (MIM# 113705) or *BRCA2* (MIM# 600185) genes [1], [2]. Carriers of these mutations have a 40% to 80% probability of developing breast cancer in their life [3] and show an increased risk of other cancers, particularly ovarian carcinoma. As a consequence, *BRCA1* and *BRCA2* genetic testing has become a widely used procedure in the clinical management of families with genetic predisposition to breast/ovarian cancer, thus allowing discrimination of at-risk mutation carriers from non-carriers, whose cancer risk can be assumed comparable to that of the general population. However, the usefulness of these molecular analyses depends on the ability to correctly distinguish truly pathogenic mutations, i.e. responsible for the increased risk of cancer, from genetic variants without clinical relevance. Most clinically relevant alterations detected in *BRCA1* and *BRCA2* are nonsense or frameshift mutations that, by introducing a premature termination codon (PTC), lead to non functional proteins. Moreover, transcripts containing PTCs are mostly subject to nonsense mediated mRNA decay (NMD) [4]. Conversely, the interpretation of other genetic variants, including missense and silent substitutions, and alterations in intronic and regulatory regions, cumulatively referred to as unclassified variants (UVs), or variants of unknown significance (VUS), is not so straightforward. As a consequence, counseling of families in which only UVs are detected is difficult, since the genetic analyses fail to unambiguously identify at-risk individuals. To increase the informativeness of genetic testing in breast/ovarian cancer families, multifactorial likelihood models for the classification of UVs have been developed and applied (reviewed in [5], [6]). These models take into account several factors. At present, these include the co-segregation of the variant with the disease in families and its co-occurrence *in trans* with a deleterious mutation in the same gene, personal and family history of cancer, histopathological tumor features, and, limited to missense mutations, the conservation across species of the affected amino acid and the nature and position of the substitution. The usefulness of integrated models is limited by the amount of data necessary to reach the required odds ratios, in favor or against causality, for reliable classification of UVs. Indeed, multifactorial likelihood methods are usually unable to classify *BRCA1* and *BRCA2* UVs detected in few families only [7]. This provides a strong rationale for the use of functional assays for the characterization of UVs under the assumption that they are highly sensitive and specific in detecting deleterious mutations.

A subgroup of UVs is represented by intronic and exonic alterations located in consensus splicing regions that are potentially pathogenic since they may lead to aberrant transcript(s), either lacking one or more exons, or even part of them, or retaining intronic sequences. Several UVs in the *BRCA1* and *BRCA2* genes with a potential consequence on mRNA splicing have been studied by cDNA analysis or reporter minigene assay. These studies show that transcript characterization is a powerful approach to correctly classify these UVs [8]–[21]. However, in some instances, different mRNA transcript patterns have been reported in association with the same mutation by diverse studies [18], [22]. This inconsistency of results between laboratories is possibly due to the different experimental protocols adopted.

Moreover, several computational programs available online have been developed to recognize the natural acceptor and donor splice sites [23]. Numerous studies have shown that these tools may be used to predict whether *BRCA1* and *BRCA2* mutations located at splice sites and adjacent regions are expected to have an effect on mRNA splicing [13]–[18], [20]–[22], [24]–[27]. Therefore, they have been proposed to be instrumental in UV classification.

In this study, we characterized by transcript analysis 24 UVs located at donor and acceptor consensus splice sites of *BRCA1* and *BRCA2,* including the nearly invariant dinucleotides at the 5′ and 3′ intron ends and adjacent nucleotides. Of the examined variants, 11 had not been previously analyzed at mRNA level, whereas 13 variants had been already examined in earlier studies. Transcript profiles observed in the latter group were compared with those previously described. In addition, we compared the experimental results with the outcome of computational analyses to evaluate the ability of different bioinformatics tools to identify deleterious splice site mutations and the nature of aberrant transcripts.

## Materials and Methods

The UVs analyzed in this study were detected following direct sequencing of all coding exons and adjacent intronic regions of *BRCA1* and *BRCA2* (GenBank no. U14680 and U43746, respectively) in index cases from families complying with the previously reported eligibility criteria for BRCA gene testing [28]. A total of 24 UVs were investigated, including 11 not previously characterized at mRNA level (10 in *BRCA1* and 1 in *BRCA2*). The variants consisted of 2 groups: the first (Group A) included 11 alterations (6 in *BRCA1* and 5 in *BRCA2*) located at nearly invariant GT/AG dinucleotides at the 5′ and 3′ intron ends, and the second (Group B) 13 alterations (9 in *BRCA1* and 4 in *BRCA2*) in the adjacent less conserved splicing regions, including the first 2 and the last 3 exonic nucleotides and the intronic regions ranging from IVS±3 to IVS+8 and IVS-12 [29].

### Ethics Statement

All subjects included in the study received genetic counseling and provided a written informed consent for BRCA gene mutation testing and for the use of their biological samples for research purposes, approved by the ethical committees of Fondazione IRCCS Istituto Nazionale Tumori and Istituto Europeo di Oncologia in Milan, and IRCCS San Martino IST- Istituto Nazionale per la Ricerca sul Cancro, Genoa.

### Cell Cultures

Epstein-Barr virus (EBV)-immortalized human lymphoblastoid cell lines (LCLs) were established from peripheral blood of UV carriers. LCLs were maintained in RPMI 1640 medium supplemented with 15% fetal bovine serum plus 1% penicillin-streptomycin. Potential degradation of unstable transcripts via NMD was prevented by growing LCLs for 6 hours in the presence of 100 µg/ml puromycin prior to RNA extraction [4]. MCF7 human breast cancer cells were cultured in DMEM medium supplemented with 10% fetal calf serum plus 1% penicillin-streptomycin. LCLs and MCF7 cells were cultured at 37°C in a humidified 5% CO_2_ atmosphere.

### RNA Extraction and Reverse Transcriptase-PCR (RT-PCR) Product Analysis

Total RNA was purified from LCLs using the Nucleospin RNA II (Macherey-Nagel). cDNA was synthesized using random primers and the ImProm-II™ Reverse Transcriptase (Promega), or gene-specific primers and SuperScript III™ Reverse Transcriptase (Invitrogen), according to the manufacturers’ protocols. For each UV studied, a specific PCR experiment was developed. Forward and reverse primers (Table S1) were designed to anneal to cDNA sequences flanking the gene region addressed by the alteration. The cDNA from a human LCL previously tested negative for *BRCA1* and *BRCA2* mutations was used as wild-type control. RT-PCR products were separated on agarose gel and visualized by ethidium bromide staining. Each UV examined was categorized as ‘normal’ or ‘spliceogenic’ (i.e., causing aberrant splicing) by comparison of the corresponding electrophoretic pattern with that of the wild-type cDNA. Altered transcript patterns were eventually confirmed by comparison with the transcript patterns observed in 10 healthy controls. Unfractionated PCR products were cleaned using ExoSAP-IT^®^ (USB Corporation) and characterized by direct sequencing. When the exact nature of each amplicon could not be assessed by the direct sequencing of PCR products, normal and aberrant bands were excised from the agarose gel, purified using the Wizard SW Gel and PCR Clean-Up System (Promega) and individually sequenced. Alternatively, the amplicons were separated by cloning into the pGEM-T vector (Promega). Recombinant plasmids were transformed into *E. Coli* (SoloPack Gold, Agilent Tecnologies) and the inserts of individual clones were sequenced. All sequence reactions were performed using the ABI PRISM® BigDye™ Terminator Cycle Sequencing kit (Applied Biosystems) and examined on an ABI 3130 Genetic Analyzer (Applied Biosystems), using the Sequencing Analysis software (Applied Biosystems).

### Assessment of Allelic Expression of Normal Transcripts

The ability of analyzed variants to synthesize normal transcripts was investigated by variant-specific PCR assays. In each assay, the primers were designed to anneal to sequences exclusive of the normal cDNA and to generate amplicons that included either the site of the exonic variant, or, if the variant was intronic, a polymorphic site for which the corresponding carrier had been previously found to be constitutionally heterozygous.

The amplification products were sequenced as previously described. In the presence of bi-allelic expression, the PCR products were cloned into the pGEM-T vector. Recombinant plasmids were transformed into *E. Coli* (SoloPack Gold, Agilent Tecnologies) and the inserts of individual clones were sequenced to quantify the relative amount of normal transcripts expressed by the wild-type and the mutant alleles.

### Pull-down Assays

Full-length *DSS1* cDNA and *BRCA2* cDNA fragments, encoding the DSS1/DNA Binding Domain (DBD) and the N-terminal region, were obtained by RT-PCR of RNA purified from wild-type and *BRCA2*-mutated LCLs, and cloned into pEGFP-C1 (DSS1) or pGEX-4T1 (BRCA2). The *BRCA2* c.8850G>T (p.Lys2950Asn) variant was inserted by direct mutagenesis into wild-type cDNA using the QuickChange XL Site-directed Mutagenesis Kit (Stratagene). Recombinant clones were verified by DNA sequencing. pGEX-4T1/BRCA2 clones were transformed into *E. Coli* strain BL21 (DE3) by electroporation. MCF7 cells were transfected with pEGFP-C1/DSS1 using FuGENE 6 Reagents (Roche Applied Science) and stable transfectants expressing green fluorescent protein (GFP)-DSS1 were obtained by selection in the presence of G418 (500 µg/ml). Single clones were checked by RT-PCR and Western blotting.

The glutathione-S-transferase (GST) tagged recombinant proteins, generated from the pGEX-4T1/BRCA2 constructs, were expressed and purified from the soluble fraction using Glutathione (GSH) Sepharose 4B beads according to the manufacturer’s protocol (Amersham Biosciences).

For DSS1 binding assays, the wild-type and mutated resin-bound GST-BRCA2 recombinant polypeptides were incubated with lysates from MCF7 GFP-DSS1 transfectants in binding buffer for 3 hours at 4°C on a rocker as described [30]. Complexes recovered from the beads were resolved on 8% SDS-PAGE gels and visualized by Coomassie blue staining or by immunoblotting with an anti-GFP antibody.

For single-stranded DNA (ssDNA) binding assays, the mutants and wild-type BRCA2 polypeptides were removed from GSH-Sepharose beads by thrombin digestion (1 U/100 µg) for 1 hour at room temperature in elution buffer (10 mM GSH in 50 mM Tris-HCl pH8.0). Free proteins were mixed with 50 µl of ssDNA agarose beads (Amersham Biosciences) and 100 µl of binding buffer (25 mM Tris-HCl pH7.5, 10% glycerol, 0.01% Triton X-100, 0.25 mM PMSF, 1 mM EDTA, 150 mM NaCl) for 2 hours at 4°C on a rocker. The supernatants were recovered and the beads washed 4 times with 300 µl of binding buffer. Equivalent amounts of supernatants (free fraction, F) and ssDNA agarose beads (bound-fraction, B) were resolved on 10% SDS-PAGE gels and visualized by Coomassie staining.

### *In silico* Splicing Analysis

Nine computational programs were investigated to verify their accuracy in correctly predicting the effect on mRNA splicing of the variants analyzed *in vitro*. These included 5 tools integrated in the Alamut application (Interactive Biosoftware, Version 2.1, Roven, France) [31], namely: Splice Site Finder (SSF) [32], MaxEntScan(MES) [33], Splice Site Prediction by Neural Network (NNSPLICE) [34], GeneSplicer (GS) [35], and Human Splicing Finder (HSF) [36], plus the following additional tools: NetGene2 (NG2) [37], [38], SpliceView (SV) [39], SplicePredictor (SP) [40], and Automated Splice Site Analyses (ASSA) [41].

Gene regions addressed by the variants under analyses were submitted to bioinformatics analyses using the human default parameter settings of the different programs. For all programs except ASSA, the splice site prediction scores (SSPSs) in the wild-type and the mutated sequences were compared and the relative percent difference was calculated as follows: [(SSPS_mut_-SSPS_wt_)/SSPS_wt_ ]x100. For ASSA, which measures the binding affinity of the spliceosome to wild-type and mutated splice sites using information theory-based values (Ri) measured in bits (where a 1 bit change represents a 2-fold change [42]), the percent difference of binding affinity in the mutated compared to the wild-type sequences was calculated as follows: [2^(Rimut-Riwt)^−1]×100.

In addition, we verified the ability of bioinformatics programs to identify the alternative splice sites that were observed in *in vitro* analyses to be activated following the destruction of the natural splice sites. For programs that were able to identify all such alternative splice sites, the sequence encompassing 500 bp upstream and downstream the natural splice site affected by the alteration was submitted to bioinformatics analyses and the SSPS and Ri patterns in the mutated sequences were analyzed.

## Results

### mRNA Transcript Analysis

The occurrence of aberrant transcripts was observed for 19 variants, including all 11 mutations of group A (Table 1), and 8 out of 13 variants of group B (Table 2).

**Table 1:** Experimentally observed effects on mRNA splicing of group A variants and predicted protein change.

Variant		SS	mRNA change observed		Allelicexpression ofnormaltranscript(s)	Predicted protein change^a^		Classificationaccording tocurrentguidelines^b^
BIC-nomenclature	HGVS-nomenclature		Description	HGVS-nomenclature		Description	HGVS-nomenclature	
*BRCA1*								
IVS7+2T>G	c.441+2T>G	D	skipping of 62 bp at the3′-end of exon 7	r.[380_441del]	mono-allelic	stop at codon 137	p.Ser127ThrfsX11	5
IVS8+2T>A^c^	c.547+2T>A	D	skipping of exon 8	r.[442_547del]	mono-allelic	stop at codon 198	p.Gln148AspfsX51	5
IVS16+1G>T	c.4986+1G>T	D	retention of 65 bp at the5′-end of intron 16	r.[4986_4987ins4986+1_4986+65; 4986+1g>u]	not assessable	stop at codon 1676	p. Met1663ValfsX14	4 or 5
IVS16−1G>A	c.4987−1G>A	A	skipping of exon 17	r.[4987_5074del]	mono-allelic	stop at codon 1672	p.Val1665SerfsX8	5
IVS20−2delA	c.5278−2delA	A	skipping of exon 21;skipping of 8 bp at the 5′-end of exon 21	r.[5278_5332del,5278_5285del]	mono-allelic	stop at codon 1774,stop at codon 1826	p.Phe1761AsnfsX14,p.Ile1760GlyfsX67	5
IVS21+1G>A	c.5332+1G>A	D	skipping of exon 21	r.[5278_5332del]	mono-allelic	stop at codon 1774	p.Phe1761AsnfsX14	5
*BRCA2*								
IVS5+1G>A	c.475+1G>A	D	skipping of exon 5	r.[426_475del]	mono-allelic	stop at codon 165	p.Pro143GlyfsX23	5
IVS5−2A>G^d^	c.476−2A>G	A	skipping of exons 6;up-regulation of Δexons 5–6 isoform	r.[ = , 476_516del,426_516del]	bi-allelic	stop at codon 168,stop at codon 154	p.Val159GlyfsX10,p.Ser142ArgfsX13	4
IVS13−2A>T^e^*	c.7008−2A>T	A	skipping of exon 14;skipping of 10 bp at 5′-end of exon 14;skipping of 246 bp at 5′-end of exon 14	r.[7008_7435del,7008_7017del,7008_7253del]	mono-allelic	stop at codon 2353,stop at codon 2363,stop at codon 3337	p.Thr2337PhefsX17,p.Thr2337AsnfsX27,p.Thr2337ValfsX1001	5
IVS21−1G>A^f^*	c.8755−1G>A	A	skipping of exon 22; skipping of exon22+51 bp at the 5′-end of exon 23	r.[ = , 8755_8953del,8755_9004del]	bi-allelic	stop at codon 2921,stop at codon 2944	p.Gly2919LeufsX3,p.Gly2919LysfsX26	4
IVS22−1delGTTinsAA^g^*	c.8954−1_8955delGTTinsAA	A	skipping of 51 bp at the 5′-end ofexon 23; skipping of exon 23	r.[8954_9004del,8954_9117del]	mono-allelic	in frame deletion of 17aa,stop at codon 2988	p.Val2985_Thr3001del,p.Val2985GlyfsX4	5

a Protein change was predicted using ExPASy Proteomics Server (http://www.expasy.ch/);

b The classification as class 5 (pathogenic) or class 4 (likely pathogenic) was based on mono- or bi-allelic expression of the normal transcript [23]. Previously characterized variants are indicated;

c [22];

d [43];

e [22], [44], [45];

f [43];

g [18]. An asterisk indicates variants for which the observed transcript pattern differed from that reported by previous studies (see Table S6). Abbreviations: SS, splice Site (D, donor; A, acceptor); BIC, Breast Cancer Information Core (http://research.nhgri.nih.gov/bic/); HGVS, Human Genetic Variation Society (http://www.hgvs.org/mutnomen).

**Table 2:** Experimentally observed effects on mRNA splicing of group B variants and predicted protein change.

Variant		SS	mRNA change observed		Allelicexpression ofnormaltranscript (s)	Predicted protein change^a^		Classificationaccording tocurrentguidelines^b^
BIC-nomenclature	HGVS-nomenclature		Description	HGVS-nomenclature		Description	HGVS-nomenclature	
*BRCA1*								
IVS3+3del AAGT	c.134+3_134+6del AAGT	D	up-regulation of Δexon3isoform	r.[81_134del]	not assessable	stop at codon 27	p.Cys27X	4 or 5
331G>A^c^	c.212G>A	D	up-regulation of Δexon5q isoform	r.[191_212del]	mono-allelic	stop at codon 64	p.Cys64X	5
IVS5−11T>G^d^	c.213−11T>G	A	retention of 59 bp atthe 3′-end of intron 5	r.[212_213ins213-59_213-1; 213-11u>g]	mono-allelic	stop at codon 81	p.Arg71SerfsX11	5
IVS8−3delT	c.548−3delT	A	none	r.[ = ]	bi-allelic	none	p. =	2
IVS9−4A>G	c.594−4A>G	A	none	r.[ = ]	bi-allelic	none	p. =	2
4216G>A	c.4097G>A	A	none	r.[4097g>a]	bi-allelic	aa change at codon 1366	p.Gly1366Asp	2
4603G>T^e^	c.4484G>T	D	skipping of exon 14	r.[4358_4484del]	mono-allelic	stop at codon 1462	p.Ala1453GlyfsX10	5
IVS16+5G>A	c.4986+5G>A	D	retention of 65 bp atthe 5′-end of intron 16	r.[4986_4987ins4986+1_4986+65; 4986+5g>a]	mono-allelic	stop at codon 1676	p. Met1663ValfsX14	5
5452A>G^f^	c.5333A>G	A	none	r.[5333a>g]	bi-allelic	aa change at codon 1778	p.Asp1778Gly	2
*BRCA2*								
859G>A^g^*	c.631G>A	D	skipping of exon 7	r.[517_631del]	mono-allelic	stop at codon 191	p.Gly173SerfsX19	5
IVS21+3G>C^h^*	c.8754+3G>C	D	retention of 46 bp atthe 5′-end of intron 21	r.[8754_8755ins8754+1_8754+46; 8754+4a>g]	mono-allelic	stop at codon 2922	p.Gly2919ValfsX4	5
9344C>T^i^*	c.9116C>T	D	none	r.[9116c>u]	bi-allelic	aa change at codon 3039	p.Pro3039Leu	2
9345G>A^j^	c.9117G>A	D	skipping of exon 23	r.[8954_9117del]	mono-allelic	stop at codon 2988	p.Val2985GlyfsX4	5

a Protein change was predicted using ExPASy Proteomics Server**.** (http://www.expasy.ch/);

b The classification as class 5 (pathogenic) or class 4 (likely pathogenic) was based on mono- or bi-allelic expression of the normal transcript [23], that of class 2 (likely neutral) on A-GVGD software prediction (http://agvgd.iarc.fr/). Previously characterized variants are indicated;

c [22], [50];

d [22], [46];

e [22], [47], [49];

f [21];

g [19], [44], [45];

h [21], [26];

i [20], [22];

j [11], [18], [22], [48]. An asterisk indicates variants for which the observed transcript pattern differed from that reported by previous studies (see Table S6). Abbreviations: SS, splice Site (D, donor; A, acceptor); BIC, Breast Cancer Information Core (http://research.nhgri.nih.gov/bic/); HGVS, Human Genetic Variation Society (http://www.hgvs.org/mutnomen/).

**Figure 1:**
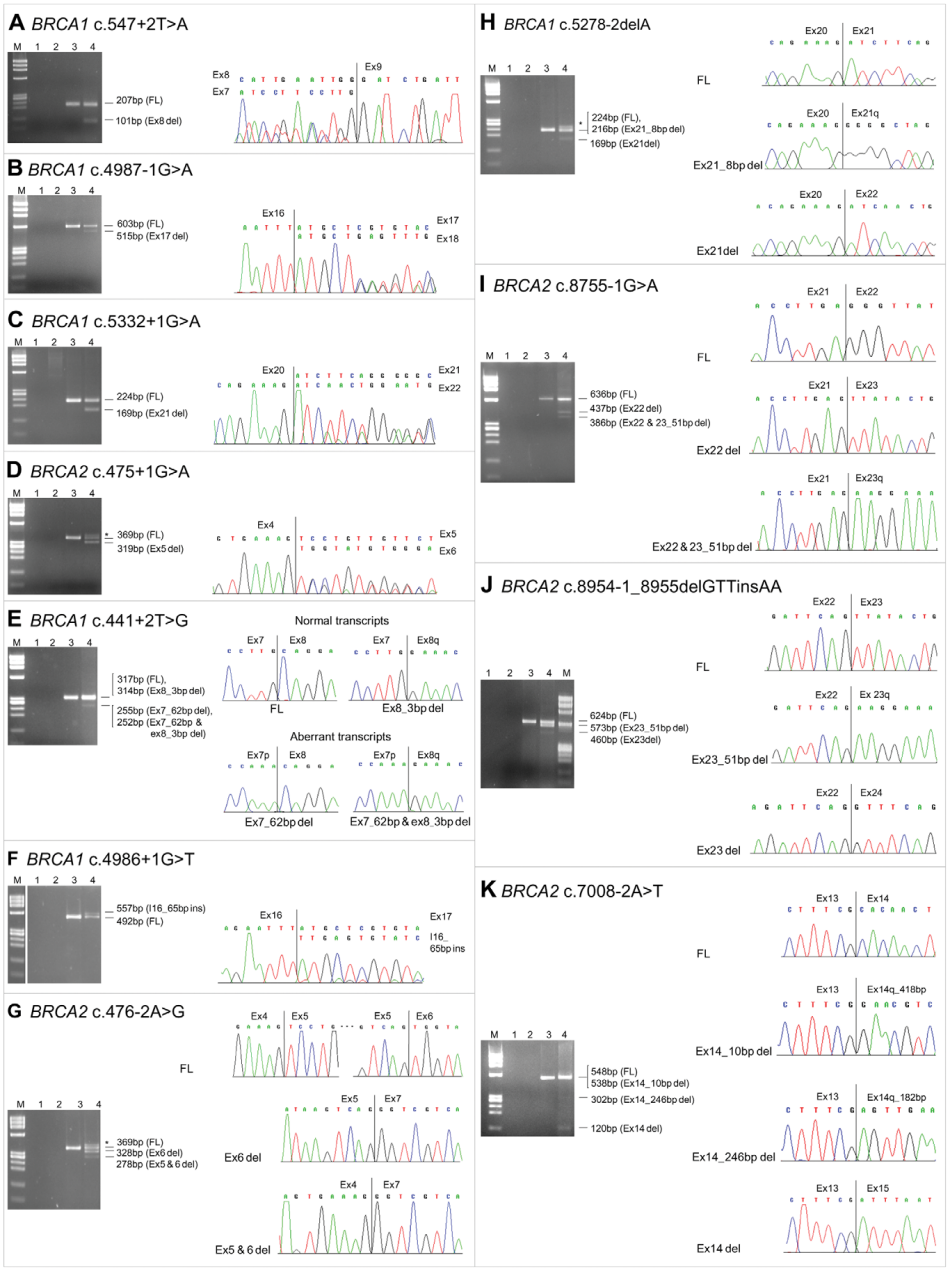
RT-PCR analyses of group A variants. For each variant, the RT-PCR products were characterized by agarose gel electrophoresis and sequencing. Gel images: lane 1, no template; lane 2, genomic DNA used as negative control of the RT-PCR reaction; lane 3, cDNA from the *BRCA1/BRCA2* wild-type LCL used as positive control; lane 4, cDNA from LCL carrying the UV. M, molecular marker (ΦX-174 HaeIII digest). The size of the full-length (FL) and aberrant transcripts are reported. Sequencing electropherogram data: (**A–D, F**) the RT-PCR products were directly sequenced; (**E, G–K**) the sequencing was performed after band excision or cloning step. (**D, G, H**) Additional bands due to improper annealing of full-length and aberrant transcripts are indicated by the asterisk. (**E**) In addition to the full-length and the Ex7_62 bp del aberrant transcript, the naturally occurring isoform lacking the first 3 bp of exon 8 (Ex8_3 bp del) was observed. Ex, exon; I, intron.

Spliceogenic mutations of group A included 5 that had been already analyzed in previous studies (c.547+2T>A in *BRCA1,* and c.476−2A>G, c.7008−2A>T, c.8755−1G>A and c.8954−1_8955delGTTinsAA in *BRCA2*) [18], [22], [43]–[45] and 6 not previously characterized (441+2T>G, c.4986+1G>T, c.4987−1G>A, c.5278−2delA, c.5332+1G>A in *BRCA1*and c.475+1G>A in *BRCA2*). In particular, *BRCA1* c.547+2T>A, c.4987−1G>A and c.5332+1G>A caused the loss of the whole exons 8, 17 and 21, respectively (Fig. 1A–C), and *BRCA2* c.475+1G>A resulted in the loss of exon 5 (Fig. 1D). In contrast, the use of alternative cryptic splice sites induced partial loss of exon 7 (62 bp at the 3′-end) for *BRCA1* c.441+2T>G (Fig. 1E) and partial retention of intron 16 (65 bp at the 5′-end) for *BRCA1* c.4986+1G>T (Fig. 1F). *BRCA2* c.476−2A>G was found to give rise to an abnormal transcript lacking exon 6 and to up-regulate the Δexons 5–6 isoform (Fig. 1G). More complex aberrant patterns were observed for the remaining spliceogenic variants. In particular, 2 aberrant transcripts were observed for *BRCA1* c.5278−2delA (one lacking exon 21 and another 8 bp at the 5′-end of exon 21) (Fig. 1H), *BRCA2* c.8755−1G>A (one lacking exon 22 and another exon 22 plus 51 bp at the 5′-end of exon 23) (Fig. 1I) and *BRCA2* c.8954−1_8955delGTTinsAA (one lacking exon 23 and another 51 bp at the 5′-end of exon 23) (Fig. 1J). Notably, *BRCA2* c.8755−1G>A and c.8954−1_8955delGTTinsAA mutations led to the activation of the same cryptic splice site in exon 23. Finally, the *BRCA2* c.7008−2A>T variant was observed to give rise to 3 aberrant transcripts, including one lacking the whole exon 14, and 2 others lacking 10 and 246 bp at the 5′-end of exon 14, respectively (Fig. 1K).

**Figure 2:**
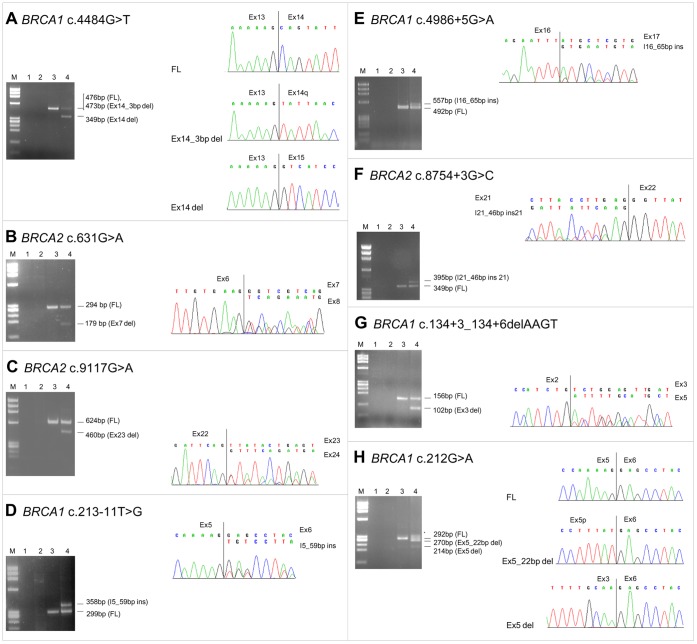
RT-PCR analyses of group B variants. For each variant, the RT-PCR products were characterized by agarose gel electrophoresis and sequencing. Gel images: lane 1, no template; lane 2, genomic DNA used as negative control of the RT-PCR reaction; lane 3, cDNA from the *BRCA1/BRCA2* wild-type LCL used as positive control; lane 4, cDNA from LCL carrying the UV. M, molecular marker (ΦX-174 HaeIII digest). The size of the full-length (FL) and aberrant transcripts are reported. Sequencing electropherogram data: (**B–G**) the RT-PCR products were directly sequenced; (**A, H**) the sequencing was performed after band excision or cloning step. (**H**) An additional band due to improper annealing of full-length and aberrant transcripts is shown by the asterisk. The Ex5del, visible in both sample and control is a naturally occurring isoform lacking exon 5. (**A**) In addition to the full-length and the Ex14del aberrant transcript, the naturally occurring isoform lacking the first 3 bp of exon 14 (Ex14_3 bp del) was observed. Ex, exon; I, intron.

Spliceogenic mutations of group B included 6 already analyzed (*BRCA1* c.212G>A, c.213−11T>G, and c.4484G>T, and *BRCA2* c.631G>A, c.8754+3G>C, and c.9117G>A) [11], [18], [19], [21], [22], [26], [44]–[50] and 2 newly characterized (c.134+3_134+6delAAGT, c.4986+5G>A in *BRCA1*). Three mutations caused the skipping of an entire exon: *BRCA1* c.4484G>T (exon 14) (Fig. 2A), *BRCA2* c.631G>A (exon 7) (Fig. 2B) and *BRCA2* c.9117G>A (exon 23) (Fig. 2C). Conversely, partial intronic retention caused by the activation of cryptic splice sites was observed for *BRCA1* c.213−11T>G (59 bp at the 3′-end of intron 5) (Fig. 2D), *BRCA1* c.4986+5G>A (65 bp at the 5′-end of intron 16) (Fig. 2E), and for *BRCA2* c.8754+3G>C (46 bp at the 5′-end of intron 21) (Fig. 2F). Finally, the *BRCA1* c.134+3_134+6delAAGT and c.212G>A variants were associated with a relevant increase, in comparison with normal controls, of the Δexon3 and Δexon5q (missing 22 bp at the 3′-end of exon 5) isoforms, respectively (Fig. 2G–H). Both isoforms contain PTCs.

To verify whether the identified spliceogenic alleles maintained the ability to synthesize wild-type mRNAs, normal transcripts were selectively amplified from the cDNAs of carriers of the investigated mutations, using variant-specific PCR assays, and sequenced. The location of PCR primers and the nucleotide changes analyzed to verify allelic expression are reported in Table S2. In 2 cases, *BRCA2* c.476−2A>G and c.8755−1G>A, cDNA sequence analyses revealed maintenance of the constitutional heterozygosity for the c.1114C>A SNP (exon 10; rs144848) and the c.9876G>A synonymous change (exon 27), respectively (data not shown), indicating expression of the normal mRNA from both the wild-type and mutated alleles. The corresponding PCR products containing the sites of heterozygosity were cloned into plasmid vectors and single recombinant clones were sequenced. A total of 23 clones were analyzed for the c.476−2A>G mutation. Of these, 20 (87%) carried the rs144848 C allele and 3 (13%) the A allele. Of the 52 clones analyzed for the 8755−1G>A mutation, 47 (90%) carried the G allele and 5 (10%) the A allele of the synonymous change.

For 11 of the 13 remaining intronic mutations cDNA sequence analyses detected hemizigosity at polymorphic sites for which the corresponding carriers were heterozygous at the genomic level (Tables 1 and 2). The occurrence of mono- or bi-allelic expression of normal transcripts could not be assessed for 2 intronic mutations (*BRCA1* c.4986+1G>T and c.134+3_134+6delAAGT) due to the lack of informative exonic polymorphisms. Finally, for all 4 spliceogenic mutations located in exons, cDNA sequencing revealed the presence of only the nucleotide corresponding to the wild-type allele.

Normal mRNA splicing was observed for the remaining 5 variants of group B, including *BRCA1* c.5333A>G and *BRCA2* c.9116C>T, already analyzed [20]–[22] and *BRCA1* c.548−3delT, c.594−4A>G, c.4097G>A, not previously characterized. To account for the possible occurrence of NMD, LCLs carrying these variants were analyzed following treatment with puromycin. No aberrant transcripts were found. In addition, sequence analyses of cDNAs, investigating the presence of the exonic variants or of constitutionally heterozygous polymorphisms, revealed bi-allelic expression in all cases.

### Functional Analysis of BRCA2 p.Val2985_Thr3001del

All 19 identified spliceogenic UVs led to PTCs, except the *BRCA2* c.8954−1_8955delGTTinsAA which resulted in the in-frame deletion of 51 nucleotides at the 5′-end of exon 23, with consequent 17-amino acids loss (p.Val2985_Thr3001del) in the DBD of the protein. In addition to ssDNA, BRCA2 DBD interacts with several proteins, including DSS1 whose binding is crucial for DNA double-strand break repair [51]. Furthermore, many *BRCA2* missense mutations, classified as deleterious by multifactorial likelihood model analysis [7], lie within this domain, emphasizing its functional role.

**Figure 3:**
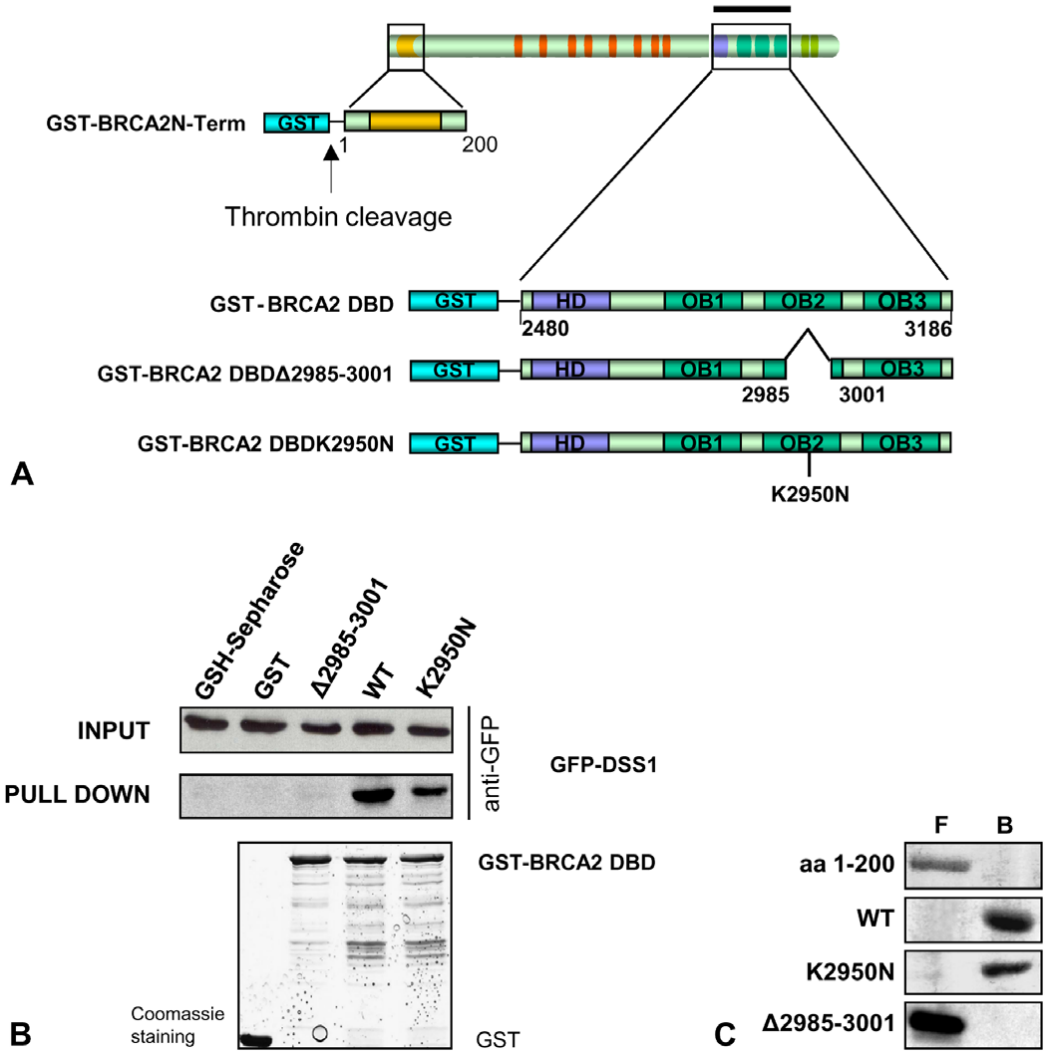
Functional analysis of BRCA2 p.Val2985_Thr3001del. (**A**) Schematic representation of GST-BRCA2 recombinant proteins. Wild-type and mutant *BRCA2* fragments, encoding the DBD and the N-terminal region, were cloned into pGEX4T1 vector to express GST-BRCA2 fusion proteins under the control of lacUV5 promoter. BRCA2 amino acid positions, helical domain (HD) and OB fold domains 1, 2, 3 (OB1, OB2, OB3) are indicated. (**B**) Interaction of wild-type and mutated BRCA2 DBD polypeptides with DSS1**.** Equivalent amounts of GST-tagged wild-type or mutated BRCA2 fusion proteins were immobilized on GSH-Sepharose beads and challenged with MCF7 lysates as a source of GFP-DSS1. Input (top panel) and pulled down (middle panel) GFP-DSS1 protein were visualized by Western blotting with anti-GFP antibody. GSH-Sepharose beads and GST protein were used as negative controls. GST-tagged recombinant proteins were visualized by Coomassie staining of the SDS-PAGE gel used in the pull-down experiment (bottom panel)**.** (**C**) Interaction of wild-type and mutated BRCA2 polypeptides with ssDNA. The mutated and wild-type peptides, removed from glutathione-agarose beads by thrombin digestion, were chromatographed on ssDNA agarose beads. A 200 amino acids N-terminal peptide was used as negative control. The free (F) and bound (B) fractions were separated, submitted to gel electrophoresis and visualized by Coomassie staining. Immunoblots were scanned using HP Scanjet G3010 Photo Scanner (Hewlett Packard).

The functional consequences of the BRCA2 p.Val2985_Thr3001del mutation were assessed by testing its effect on DBD binding to DSS1 and ssDNA. Wild-type and mutant resin-bound GST-BRCA2 DBD polypeptides (Fig. 3A) were used as bait in pull-down experiments against extracts from MCF7 GFP-DSS1 transfectants, and the extent of DSS1 binding was evaluated by Western blotting using an anti-GFP antibody. DSS1 protein was found to interact efficiently with BRCA2 DBD wild-type and BRCA2 DBD carrying a variant (p.Lys2950Asn) classified as clinically neutral [7], while BRCA2 DBD Val2985_Thr3001del mutant failed to interact with DSS1 (Fig. 3B).

To evaluate the affinity of the p.Val2985_Thr3001del mutant for ssDNA, both wild-type and mutated BRCA2 DBD polypeptides, along with a BRCA2 200-aa N-terminal polypeptide, as negative control, were cleaved from the GST-agarose beads by thrombin digestion and chromatographed on ssDNA agarose beads. The pellets, representing the ssDNA-bound fraction, and the accompanying supernatants were analyzed separately by gel electrophoresis and stained with Coomassie dye. The BRCA2 wild-type and p.Lys2950Asn polypeptides were both recovered in the ssDNA agarose bead fraction (bound fraction, B), whereas the N-terminal fragment and the p.Val2985_Thr3001del mutant were completely recovered in the supernatant fraction (free fraction, F) (Fig. 3C). These results indicate that the c.8954−1_8955delGTTinsAA mutation, causing in-frame 17-aa deletion in the DBD domain, abrogates the ability of BRCA2 to bind the DSS1 protein and its affinity for ssDNA.

### *In silico* Splicing Analysis

We pursued the study, examining the ability of *in silico* tools to discriminate between spliceogenic and non-spliceogenic variants. Since all group A variants were found to be spliceogenic *in vitro*, this analysis was restricted to group B variants. Computed values (SSPSs for all programs except ASSA, and Ri for ASSA) in wild-type and mutated sequences are reported in Table S3. We observed that most programs failed to recognize the presence of one or more natural splice sites in the wild-type sequences of *BRCA1* and *BRCA2*, using default settings. Therefore, these programs could not be used to evaluate the effect of variants located in these unrecognized sites (non informative analysis). Only 3 programs (MES, HSF and ASSA) were found to identify all investigated splice sites.

Then, for each program we verified the smallest SSPS/Ri percent decrease observed for a spliceogenic mutation. This value (varying from 4.1% for HSF to 100% for GS and SP) was assumed as the minimal SSPS/Ri difference predictive of a spliceogenic mutation (Table 3). This was done in order to set to 100% the sensitivity of *in silico* analyses in identifying mRNA affecting variants. Eventually, for each program we calculated the rate of false positive analyses (i.e., the number of variants incorrectly classified as spliceogenic on the total number of true non spliceogenic variants) as a measure of the specificity of their predictions. Considering informative analyses only, the rate of false positive analyses ranged from 0% for SSF, GS, HSF, SV and ASSA to 50% for NNSPLICE and NG2 (Table 3).

**Table 3:** *In silico* predicted effect of group B variants and comparison with experimental results.

Variant HGVS-nomenclature	Aberrant mRNAs	SSF	MES	NNSPLICE	GS	HSF	NG2	SV	SP	ASSA
*BRCA1*										
c.134+3_134+6del AAGT	YES	−100%; S (C)	−100%; S (C)	−100%; S (C)	−100%; S (C)	−25.7%; S (C)	−100%; S (C)	−100%; S (C)	−100%; S (C)	−99.7%; S (C)
c.212G>A	YES	−100%; S (C)	−81.6%; S (C)	−100%; S (C)	−100%; S (C)	−13.5%; S (C)	−100%; S (C)	−100%; S (C)	−100%; S (C)	−88.3%; S (C)
c.213−11T>G	YES	−100%; S (C)	−100%; S (C)			−4.1%; S (C)	−100%; S (C)	−100%; S (C)		−81.05%; S (C)
c.548−3delT	NO		−39.8; S (D)			−2.3%; NS (C)			+0.6%; NS (C)	0.0%; NS (C)
c.594−4A>G	NO	0.0%; NS (C)	+1.5; NS (C)	−1.6%; NS (C)	+13.4; NS(C)	−0.1%; NS (C)	−100%; S (D)	0.0%; NS (C)	+0.1%; NS (C)	+7.2%; NS (C)
c.4097G>A	NO	−4.4%; NS (C)	−23.4%; NS (C)	−13.9%; S (D)	−12.3%; NS (C)	−3.8%; NS (C)		−2.4%; NS (C)	−0.1%; NS (C)	−46.4%; NS (C)
c.4484G>T	YES	−13.4%; S (C)	−47.4%; S (C)	−9.8%; S (C)	−100%; S (C)	−11.2%; S (C)	−44.4%; S (C)	−6.7%; S (C)	−100%; S (C)	−89.8%; S (C)
c.4986+5G>A	YES	−100%; S (C)	−100%; S (C)	−100%; S (C)		−15.0%; S (C)	−100%; S (C)	−100%; S (C)		−91.2%; S (C)
c.5333A>G	NO	+5.4%; NS (C)	+12.3%; NS (C)	+32.5%; NS (C)	+17.7%; NS(C)	+3.9%; NS (C)	0.0%; NS (C)	+2.6%; NS (C)	0.0%; NS (C)	+86.6%; NS (C)
*BRCA2*										
c.631G>A	YES	−100%; S (C)	−100%; S (C)	−100%; S (C)		−12.7%; S (C)		−100%; S (C)		−87.5%; S (C)
c.8754+3G>C	YES	−6.3%; S (C)	−31.5; S (C)	−35.5%; S (C)	−100%; S (C)	−7.4%; S (C)	−29.0%; S (C)	−8.0%; S (C)	−100%; S (C)	−95.3%; S (C)
c.9116C>T	NO		−43.6%; S (D)	−100%; S (D)		0.0%; NS (C)		0.0%; NS (C)	−100%; S (D)	−6.7%; NS (C)
c.9117G>A	YES		−100%; S (C)	−100%; S (C)		−14.7%; S (C)		−100%; S (C)	−100%; S (C)	−87.5%; S (C)
False positive analyses rate (%)		0.0 (0/3)	40.0 (2/5)	50.0% (2/4)	0.0 (0/3)	0.0 (0/5)	50.0 (1/2)	0.0 (0/4)	20.0 (1/5)	0.0 (0/5)

For all computational program except ASSA, the relative percent differences of the splice site prediction scores (SSPSs) in the wild-type and the mutated sequences are reported. For ASSA, which uses the information theory-base values (Ri), the percent differences of binding affinity in the mutated compared to the wild-type sequences are reported. Empty cells indicates natural splice site not recognized by the indicated programs, *In silico* analyses predicting spliceogenic (S) or non spliceogenic (NS) variants according to the described procedure (see text) are indicated. (C) indicates *in silico* predictions concordant with *in vitro* data; (D), discordant predictions. Abbreviations: HGVS, Human Genetic Variation Society (http://www.hgvs.org/mutnomen/).

The SSPS/Ri values in the wild-type and mutated sequences of the alternative splice sites that were observed *in vitro* to be used following the inactivation of the natural splice sites are reported in Table S4. Only 3 programs (MES, HSF and ASSA) were found to recognize all such alternative sites in the mutated sequences, either as newly created or cryptic sites (i.e. either not predicted or already predicted in the wild-type sequence, respectively). Limited to these programs, we examined the SSPS/Ri patterns in the mutated sequences spanning ±500 bp from the natural splice site. We found that in most cases the alternative splice sites actually used were not those with the highest SSPS/Ri in the considered region, or those closest to the abrogated natural splice site. Moreover, the simultaneous occurrence observed *in vitro* for some of the investigated mutations (*BRCA1* c.5278−2delA and *BRCA2* c.7008−2A>T; c.8755−1G>A; c.8954−1_8955delGTTinsAA) of more than one aberrant transcript could not be immediately inferred from the computed SSPS/Ri patterns (Table S5).

## Discussion

In this study we molecularly characterized 24 UVs in the *BRCA1* and *BRCA2* genes with potential effect at mRNA level. A total of 19 spliceogenic mutations were identified. These included all 11 variants located at invariant dinucleotides at the 5′ and 3′ intron ends, as expected, and 8 out of 13 UVs in less conserved positions of splicing regions. Sixteen mutations led to the synthesis of aberrant transcripts containing PTCs, 2 (*BRCA1* c.134+3_134+6delAAGT and c.212G>A) to the up-regulation of naturally occurring PTC-containing isoforms, and one (*BRCA2* c.8954−1_8955delGTTinsAA) to the in-frame deletion of 51 nucleotides at the 5′-end of exon 23, within the region coding for the DBD, a critical functional domain of the BRCA2 protein. Functional analyses revealed that the latter alteration caused the loss in the mutant protein of the ability to bind the partner protein DSS1 and ssDNA. Based on these observations, all spliceogenic mutations were classified as pathogenic or likely pathogenic, according to current guidelines for the interpretation of the results of *in vitro* splicing analyses [23]. These guidelines adopt the 5-class classification criteria proposed by Plon et al. [52], and classify spliceogenic mutations as of class 5 (probability of being pathogenic >99%) or of class 4 (probability of being pathogenic = 95%–99%), depending on the relative amount of aberrant transcripts. Following this scheme, 15 mutations for which only expression of aberrant transcripts was observed, were considered of class 5, whereas the 2 mutations that maintained the ability to express normal in addition to aberrant transcripts were provisionally categorized as of class 4. To assess the relative amount of normal and aberrant transcripts expressed by these alleles, additional quantitative analyses are required. For the remaining 2 spliceogenic mutations the distinction in either class 4 or 5 could not be made due to the inability to assess allelic specific expression of the normal mRNA (Tables 1 and 2).

It must be remarked that a recent study, based on the analysis of LCL mRNA, reported 4 spliceogenic BRCA gene mutations introducing PTCs that were classified as uncertain or likely neutral by multifactorial likelihood analyses [17]. Although it is likely, as the authors of the study reported, that this discrepancy depended on a reduced performance of the multifactorial analyses, due either to a paucity of information and/or the use of non specific prior probability of pathogenicity for the variants analyzed, these data suggest that the mutation effect detected in blood cells may not necessarily reflect that occurring in at-risk tissues, such as breast and ovarian epithelium. Another possible explanation for the inconsistency between the outcome of *in vitro* splicing analyses and that of multifactorial models is the occurrence of spliceogenic mutations that maintain the ability to synthesize a normal in addition to an aberrant mRNA [13], [14], [16], [19], [25], [26], [53]. These mutations may have an impact on cancer risk different from that of fully inactivating alterations. As mentioned above, we detected 2 such mutations (BRCA2 c.476−2A>G and c.8755−1G>A). However, quantitative analyses indicated that in both cases the contribution of the mutated allele to the total amount of normal mRNA was small. Assuming that most normal mRNA transcripts derive from the wild-type allele, we found that only approximately 10% originated from the mutated allele. Both the above mutations were detected in a single family each, and no sufficient data were available for a reliable classification using multifactorial models. It is interesting to note that, although splice site mutations producing both normal and aberrant transcript would be expected to be prevalently, if not exclusively, located in less conserved regions, both identified ‘leaky’ mutations were localized at the nearly invariant dinucleotides at the 5′ and 3′ intron ends. However, we could not formally rule out that expression of normal transcripts occurred also for other examined spliceogenic mutations, due to the relatively limited sensitivity of sequencing analyses in assessing allelic specific expression.

Of the 5 non spliceogenic variants, 2 were intronic and 3 introduced missense changes (p.Gly1366Asp and p.Asp1778Gly in *BRCA1* and p.Pro3039Leu in *BRCA2*). For all the latter substitutions, the Align-GVGD algorithm [54] predicted a prior probability of pathogenicity of 1%. Therefore, following current guidelines [23], all non spliceogenic variants were classified as likely non pathogenic (class 2, probability of pathogenicity = 0,1%–4,9%). This classification was in agreement with additional evidence from previous studies. In particular, *BRCA1* p.Asp1778Gly located in the C-terminus transcriptional activation BRCT domain of the gene was predicted as neutral by 3 computational supervised learning algorithms based on features describing evolutionary conservation, impact of mutation on protein structure, and amino acid residue [55]. This prediction has been recently confirmed by a comprehensive analysis using biochemical and cell-based transcriptional assays [56]. In addition, the presence of the variant was not detected in the proband’s affected mother. Finally, the *BRCA2* p.Pro3039Leu has been classified as neutral using a bioinformatics approach integrating information about protein sequence, conservation and structure in a likelihood ratio [57].

For 8 of the 13 variants that had been already investigated at the cDNA level, our findings were consistent with those of earlier reports, while for the remaining 5 variants (all spliceogenic) the observed transcript patterns differed from those described by previous studies (Table S6). This was possibly due to the different experimental protocols that were used, suggesting that differences may occur in the ability of *in vitro* analyses to detect mRNA transcripts, particularly those expressed at low level. Another potential source of inconsistency might be the use of different types of biological samples. Although no discrepancies emerged in the classification of the examined variants as spliceogenic or non-spliceogenic when comparing our data with those of previous studies, our observations emphasize the need of developing standardized methods for *in vitro* characterization of UVs through gene transcript analyses, particularly when the outcomes of these analyses are used to counsel carriers of variants at splice sites.

In previous studies, bioinformatics analyses have been proposed as a first step to select variants predicted to affect mRNA splicing and, in particular, those located outside the nearly invariant dinucleotides at the 5′ and 3′ intron ends [14]–[16], [20], [22]. To further verify the reliability and the usefulness of these programs for *a priori* selection of spliceogenic UVs, we compared the computational splice-site predictions obtained from 9 commonly used programs with the experimental results derived from cDNA analyses. Consistent with previous reports [14], [16], [17], [20]–[22], [25], [26], we found that most tested programs showed an incomplete informativeness, i.e. were not able to recognize all natural splice sites affected by the variants under analyses. Thus, the effect of nucleotide substitutions at these sites could not be subsequently computed, limiting the usefulness of these programs. In our analysis only 3 programs (MES, HSF and ASSA) exhibited 100% informativeness.

While the performance of a selective process is usually measured in terms of accuracy, i.e., the optimal compromise between sensitivity and specificity, it must be considered that UV classification in cancer predisposing genes is manly carried out for clinical purposes, i.e., to define risk estimates in carriers of such variants [52]. Along this line, we reasoned that a mandatory pre-requirement of the procedures for *BRCA1* and *BRCA2* variant selection for transcript characterization is 100% sensitivity. Therefore, in our study, we considered that a spliceogenic effect was predicted when an *in silico* analysis measured a relative decrease of the SSPS/Ri values (of the natural splice site in the mutated compared to the wild-type sequence) higher than the lowest detected in the presence of an *in vitro* verified spliceogenic mutation. Based on this assumption, we eventually verified the specificity, measured as the rate of false positive predictions, of each program. In our hands, this was found to be equal to 100%, i.e. no false positive prediction, for 5 programs: SSF, GS, HSF, SV and ASSA. In a general evaluation, the programs that performed better were HSF and ASSA, the only exhibiting 100% informativeness and 100% specificity.

The knowledge of the precise nature of aberrant transcripts is crucial for the assessment of the pathogenicity of spliceogenic mutations. For example, variant alleles producing transcripts carrying in-frame deletions not disrupting known functional domains are currently classified as of unknown clinical significance [23] and some of them might actually be clinically neutral. This is supported by the observation that the *BRCA2* c.6853A>G variant, resulting in increased exclusion of exon 12, is phenotypically indistinguishable from an allele with exon 12 deleted and wild-type *BRCA2* in functional analyses using allelic complementation in Brca2-null mouse embryonic stem cells [58]. Therefore, it is important to ascertain whether a spliceogenic mutation, in addition to abolishing the recognition of a natural splice site, leads to the creation of novel splice sites or the activation of cryptic ones. As already discussed, in this study the usage of alternative splice sites were observed in a relevant fraction of ascertained spliceogenic variants (10/19  =  42%). We sought to verify to which extent computational programs are able to predict such occurrences. We found that only 3 programs (MES, HSF and ASSA) recognized all experimentally ascertained alternative splice sites. However, these programs also detected other putative cryptic splice sites in the vicinity of the abolished naturally-occurring splice sites and, consistent with a previous report [21], we were unable to derive simple criteria, based on the outcomes of the *in silico* analyses, for the prediction of the specific alternatively used splice sites. On the other hand, it is also possible that some of the cryptic sites predicted *in silico* could be activated in mutant samples, but the corresponding aberrant transcripts were not observed *in vitro* due to a limited sensitivity of the detection method we used.

### Conclusions

Our study provides further evidences that *in silico* tools may be used for the ascertainment of splice site variants to be submitted to *in vitro* analyses. We performed a comparative analysis of 9 freely-available computational programs, and found that those that performed better in identifying variants affecting RNA splicing, under our analytical scheme, were HSF and ASSA. However, *in vitro* analyses remain mandatory for the characterization of the exact nature of aberrant transcripts. Wider surveys within the frame of large collaborative consortia, such as the recently established ‘Evidence-based Network for the Interpretation of Germline Mutant Alleles’ (ENIGMA) [59], are looked-for, in order to define the more effective protocols for the use of bioinformatics analyses in the ascertainment of spliceogenic mutations.

## Supporting Information

Table S1 Primers used for mRNA transcripts analysis.
(DOCX)

Table S2 Nucleotide changes and primers used to assess the allelic expression of normal transcripts in carriers of analyzed variants.
(DOCX)

Table S3 *In silico* analyses of group B variants.
(DOCX)

Table S4 *In silico* analyses of spliceogenic variants leading to the activation/creation of alternative splice sites.
(DOCX)

Table S5 Location of putative splice sites and corresponding SSPS/Ri values identified by three bioinformatics programs in the *BRCA1* and *BRCA2* gene regions spanning the natural splice sites affected by the indicated spliceogenic mutations and adjacent sequences.
(DOCX)

Table S6 Spliceogenic variants for which different transcripts patterns were observed in the present compared with previous studies and experimental details.
(DOCX)

## References

[1] Stratton MR, Rahman N (2008) The emerging landscape of breast cancer susceptibility. Nat Genet 40: 17–22. 18163131 10.1038/ng.2007.53

[2] Mavaddat N, Antoniou AC, Easton DF, Garcia-Closas M (2010) Genetic susceptibility to breast cancer. Mol Oncol 4: 174–91. 20542480 10.1016/j.molonc.2010.04.011 PMC5527934

[3] Antoniou A, Pharoah PD, Narod S, Risch HA, Eyfjord JE, et al (2003) Average risks of breast and ovarian cancer associated with BRCA1 or BRCA2 mutations detected in case Series unselected for family history: a combined analysis of 22 studies. Am J Hum Genet 72: 1117–30. 12677558 10.1086/375033 PMC1180265

[4] Perrin-Vidoz L, Sinilnikova OM, Stoppa-Lyonnet D, Lenoir GM, Mazoyer S (2002) The nonsense-mediated mRNA decay pathway triggers degradation of most BRCA1 mRNAs bearing premature termination codons. Hum Mol Genet 11: 2805–14. 12393792 10.1093/hmg/11.23.2805

[5] Spurdle AB (2010) Clinical relevance of rare germline sequence variants in cancer genes: evolution and application of classification models. Curr Opin Genet Dev 20: 315–23. 20456937 10.1016/j.gde.2010.03.009

[6] Lindor NM, Guidugli L, Wang X, Vallee MP, Monteiro AN, et al (2012) A review of a multifactorial probability-based model for classification of BRCA1 and BRCA2 variants of uncertain significance (VUS). Hum Mutat 33: 8–21. 21990134 10.1002/humu.21627 PMC3242438

[7] Easton DF, Deffenbaugh AM, Pruss D, Frye C, Wenstrup RJ, et al (2007) A systematic genetic assessment of 1,433 sequence variants of unknown clinical significance in the BRCA1 and BRCA2 breast cancer-predisposition genes. Am J Hum Genet 81: 873–83. 17924331 10.1086/521032 PMC2265654

[8] Campos B, Diez O, Domenech M, Baena M, Balmana J, et al (2003) RNA analysis of eight BRCA1 and BRCA2 unclassified variants identified in breast/ovarian cancer families from Spain. Hum Mutat 22: 337. 10.1002/humu.9176 12955719

[9] Claes K, Poppe B, Machackova E, Coene I, Foretova L, et al (2003) Differentiating pathogenic mutations from polymorphic alterations in the splice sites of BRCA1 and BRCA2. Genes Chromosomes Cancer 37: 314–20. 12759930 10.1002/gcc.10221

[10] Tesoriero AA, Wong EM, Jenkins MA, Hopper JL, Brown MA, et al (2005) Molecular characterization and cancer risk associated with BRCA1 and BRCA2 splice site variants identified in multiple-case breast cancer families. Hum Mutat 26: 495. 16211554 10.1002/humu.9379

[11] Bonatti F, Pepe C, Tancredi M, Lombardi G, Aretini P, et al (2006) RNA-based analysis of BRCA1 and BRCA2 gene alterations. Cancer Genet Cytogenet 170: 93–101. 17011978 10.1016/j.cancergencyto.2006.05.005

[12] Chen X, Truong TT, Weaver J, Bove BA, Cattie K, et al (2006) Intronic alterations in BRCA1 and BRCA2: effect on mRNA splicing fidelity and expression. Hum Mutat 27: 427–35. 16619214 10.1002/humu.20319

[13] Bonnet C, Krieger S, Vezain M, Rousselin A, Tournier I, et al (2008) Screening BRCA1 and BRCA2 unclassified variants for splicing mutations using reverse transcription PCR on patient RNA and an ex vivo assay based on a splicing reporter minigene. J Med Genet 45: 438–46. 18424508 10.1136/jmg.2007.056895

[14] Vreeswijk MP, Kraan JN, van der Klift HM, Vink GR, Cornelisse CJ, et al (2009) Intronic variants in BRCA1 and BRCA2 that affect RNA splicing can be reliably selected by splice-site prediction programs. Hum Mutat 30: 107–14. 18693280 10.1002/humu.20811

[15] Sanz DJ, Acedo A, Infante M, Duran M, Perez-Cabornero L, et al (2010) A high proportion of DNA variants of BRCA1 and BRCA2 is associated with aberrant splicing in breast/ovarian cancer patients. Clin Cancer Res 16: 1957–67. 20215541 10.1158/1078-0432.CCR-09-2564

[16] Thery JC, Krieger S, Gaildrat P, Revillion F, Buisine MP, et al (2011) Contribution of bioinformatics predictions and functional splicing assays to the interpretation of unclassified variants of the BRCA genes. Eur J Hum Genet 19: 1052–8. 21673748 10.1038/ejhg.2011.100 PMC3190263

[17] Whiley PJ, Guidugli L, Walker LC, Healey S, Thompson BA, et al (2011) Splicing and multifactorial analysis of intronic BRCA1 and BRCA2 sequence variants identifies clinically significant splicing aberrations up to 12 nucleotides from the intron/exon boundary. Hum Mutat 32: 678–87. 21394826 10.1002/humu.21495 PMC4340479

[18] Acedo A, Sanz DJ, Duran M, Infante M, Perez-Cabornero L, et al (2012) Comprehensive splicing functional analysis of DNA variants of the BRCA2 gene by hybrid minigenes. Breast Cancer Res 14: R87. 22632462 10.1186/bcr3202 PMC3446350

[19] Gaildrat P, Krieger S, Di GD, Abdat J, Revillion F, et al (2012) Multiple sequence variants of BRCA2 exon 7 alter splicing regulation. J Med Genet 49: 609–17. 22962691 10.1136/jmedgenet-2012-100965

[20] Menendez M, Castellsague J, Mirete M, Pros E, Feliubadalo L, et al (2012) Assessing the RNA effect of 26 DNA variants in the BRCA1 and BRCA2 genes. Breast Cancer Res Treat 132: 979–92. 21735045 10.1007/s10549-011-1661-5

[21] Thomassen M, Blanco A, Montagna M, Hansen TV, Pedersen IS, et al (2012) Characterization of BRCA1 and BRCA2 splicing variants: a collaborative report by ENIGMA consortium members. Breast Cancer Res Treat 132: 1009–23. 21769658 10.1007/s10549-011-1674-0

[22] Houdayer C, Caux-Moncoutier V, Krieger S, Barrois M, Bonnet F, et al (2012) Guidelines for splicing analysis in molecular diagnosis derived from a set of 327 combined in silico/in vitro studies on BRCA1 and BRCA2 variants. Hum Mutat 33: 1228–38. 22505045 10.1002/humu.22101

[23] Spurdle AB, Couch FJ, Hogervorst FB, Radice P, Sinilnikova OM (2008) Prediction and assessment of splicing alterations: implications for clinical testing. Hum Mutat 29: 1304–13. 18951448 10.1002/humu.20901 PMC2832470

[24] Sharp A, Pichert G, Lucassen A, Eccles D (2004) RNA analysis reveals splicing mutations and loss of expression defects in MLH1 and BRCA1. Hum Mutat 24: 272. 10.1002/humu.9267 15300854

[25] Walker LC, Whiley PJ, Couch FJ, Farrugia DJ, Healey S, et al (2010) Detection of splicing aberrations caused by BRCA1 and BRCA2 sequence variants encoding missense substitutions: implications for prediction of pathogenicity. Hum Mutat 31: E1484–E1505. 20513136 10.1002/humu.21267 PMC3021973

[26] Brandao RD, van RK, Tserpelis D, Garcia EG, Blok MJ (2011) Characterisation of unclassified variants in the BRCA1/2 genes with a putative effect on splicing. Breast Cancer Res Treat 129: 971–82. 21638052 10.1007/s10549-011-1599-7

[27] Mucaki EJ, Ainsworth P, Rogan PK (2011) Comprehensive prediction of mRNA splicing effects of BRCA1 and BRCA2 variants. Hum Mutat 32: 735–42. 21523855 10.1002/humu.21513

[28] Manoukian S, Peissel B, Pensotti V, Barile M, Cortesi L, et al (2007) Germline mutations of TP53 and BRCA2 genes in breast cancer/sarcoma families. Eur J Cancer 43: 601–6. 17224268 10.1016/j.ejca.2006.09.024

[29] Cartegni L, Chew SL, Krainer AR (2002) Listening to silence and understanding nonsense: exonic mutations that affect splicing. Nat Rev Genet 3: 285–98. 11967553 10.1038/nrg775

[30] Kaelin WG Jr, Pallas DC, DeCaprio JA, Kaye FJ, Livingston DM (1991) Identification of cellular proteins that can interact specifically with the T/E1A-binding region of the retinoblastoma gene product. Cell 64: 521–32. 1825028 10.1016/0092-8674(91)90236-r

[31] Houdayer C (2011) In silico prediction of splice-affecting nucleotide variants. Methods Mol Biol 760: 269–81. 21780003 10.1007/978-1-61779-176-5_17

[32] Shapiro MB, Senapathy P (1987) RNA splice junctions of different classes of eukaryotes: sequence statistics and functional implications in gene expression. Nucleic Acids Res 15: 7155–74. 3658675 10.1093/nar/15.17.7155 PMC306199

[33] Yeo G, Burge CB (2004) Maximum entropy modeling of short sequence motifs with applications to RNA splicing signals. J Comput Biol 11: 377–94. 15285897 10.1089/1066527041410418

[34] Reese MG, Eeckman FH, Kulp D, Haussler D (1997) Improved splice site detection in Genie. J Comput Biol 4: 311–23. 9278062 10.1089/cmb.1997.4.311

[35] Pertea M, Lin X, Salzberg SL (2001) GeneSplicer: a new computational method for splice site prediction. Nucleic Acids Res 29: 1185–90. 11222768 10.1093/nar/29.5.1185 PMC29713

[36] Desmet FO, Hamroun D, Lalande M, Collod-Beroud G, Claustres M, et al (2009) Human Splicing Finder: an online bioinformatics tool to predict splicing signals. Nucleic Acids Res 37: e67. 19339519 10.1093/nar/gkp215 PMC2685110

[37] Brunak S, Engelbrecht J, Knudsen S (1991) Prediction of human mRNA donor and acceptor sites from the DNA sequence. J Mol Biol 220: 49–65. 2067018 10.1016/0022-2836(91)90380-o

[38] Hebsgaard SM, Korning PG, Tolstrup N, Engelbrecht J, Rouze P, et al (1996) Splice site prediction in Arabidopsis thaliana pre-mRNA by combining local and global sequence information. Nucleic Acids Res 24: 3439–52. 8811101 10.1093/nar/24.17.3439 PMC146109

[39] Rogozin IB, Milanesi L (1997) Analysis of donor splice sites in different eukaryotic organisms. J Mol Evol 45: 50–9. 9211734 10.1007/pl00006200

[40] Brendel V, Xing L, Zhu W (2004) Gene structure prediction from consensus spliced alignment of multiple ESTs matching the same genomic locus. Bioinformatics 20: 1157–69. 14764557 10.1093/bioinformatics/bth058

[41] Nalla VK, Rogan PK (2005) Automated splicing mutation analysis by information theory. Hum Mutat 25: 334–42. 15776446 10.1002/humu.20151

[42] Schneider TD (1997) Sequence walkers: a graphical method to display how binding proteins interact with DNA or RNA sequences. Nucleic Acids Res 25: 4408–15. 9336476 10.1093/nar/25.21.4408 PMC147041

[43] Machackova E, Foretova L, Lukesova M, Vasickova P, Navratilova M, et al (2008) Spectrum and characterisation of BRCA1 and BRCA2 deleterious mutations in high-risk Czech patients with breast and/or ovarian cancer. BMC Cancer 20 8: 140. 10.1186/1471-2407-8-140 PMC2413254 18489799

[44] Colombo M, Ripamonti CB, Pensotti V, Foglia C, Peissel B, et al (2009) An unusual BRCA2 allele carrying two splice site mutations. Ann Oncol 20: 1143–4. 19423647 10.1093/annonc/mdp241

[45] Pensabene M, Spagnoletti I, Capuano I, Condello C, Pepe S, et al (2009) Two mutations of BRCA2 gene at exon and splicing site in a woman who underwent oncogenetic counseling. Ann Oncol 20: 874–8. 19179552 10.1093/annonc/mdn724

[46] Friedman LS, Ostermeyer EA, Szabo CI, Dowd P, Lynch ED, et al (1994) Confirmation of BRCA1 by analysis of germline mutations linked to breast and ovarian cancer in ten families. Nat Genet 8: 399–404. 7894493 10.1038/ng1294-399

[47] Ozcelik H, Nedelcu R, Chan VW, Shi XH, Murphy J, et al (1999) Mutation in the coding region of the BRCA1 gene leads to aberrant splicing of the transcript. Hum Mutat 14: 540–1. 10571952 10.1002/(SICI)1098-1004(199912)14:6<540::AID-HUMU13>3.0.CO;2-C

[48] Peelen T, van VM, Bosch A, Bignell G, Vasen HF, et al (2000) Screening for BRCA2 mutations in 81 Dutch breast-ovarian cancer families. Br J Cancer 82: 151–6. 10638982 10.1054/bjoc.1999.0892 PMC2363204

[49] Yang Y, Swaminathan S, Martin BK, Sharan SK (2003) Aberrant splicing induced by missense mutations in BRCA1: clues from a humanized mouse model. Hum Mol Genet 12: 2121–31. 12915465 10.1093/hmg/ddg222

[50] Zhang L, Chen L, Bacares R, Ruggeri JM, Somar J, et al (2011) BRCA1 R71K missense mutation contributes to cancer predisposition by increasing alternative transcript levels. Breast Cancer Res Treat 130: 1051–6. 21863257 10.1007/s10549-011-1732-7

[51] Yang H, Jeffrey PD, Miller J, Kinnucan E, Sun Y, et al (2002) BRCA2 function in DNA binding and recombination from a BRCA2-DSS1-ssDNA structure. Science 297: 1837–48. 12228710 10.1126/science.297.5588.1837

[52] Plon SE, Eccles DM, Easton D, Foulkes WD, Genuardi M, et al (2008) Sequence variant classification and reporting: recommendations for improving the interpretation of cancer susceptibility genetic test results. Hum Mutat 29: 1282–91. 18951446 10.1002/humu.20880 PMC3075918

[53] Zhang L, Bacares R, Boyar S, Hudis C, Nafa K, et al (2009) cDNA analysis demonstrates that the BRCA2 intronic variant IVS4–12del5 is a deleterious mutation. Mutat Res 663: 84–9. 19070627 10.1016/j.mrfmmm.2008.11.010

[54] Tavtigian SV, Deffenbaugh AM, Yin l, Judkins T, Scholl T, et al (2006) Comprehensive statistical study of 452 BRCA1 missense substitutions with classification of eight recurrent substitutions as neutral. J Med Genet 43: 295–305. 16014699 10.1136/jmg.2005.033878 PMC2563222

[55] Karchin R, Monteiro AN, Tavtigian SV, Carvalho MA, Sali A (2007) Functional impact of missense variants in BRCA1 predicted by supervised learning. PLoS Comput Biol 3: e26. 17305420 10.1371/journal.pcbi.0030026 PMC1797820

[56] Lee MS, Green R, Marsillac SM, Coquelle N, Williams RS, et al (2010) Comprehensive analysis of missense variations in the BRCT domain of BRCA1 by structural and functional assays. Cancer Res 70: 4880–90. 20516115 10.1158/0008-5472.CAN-09-4563 PMC3040717

[57] Karchin R, Agarwal M, Sali A, Couch F, Beattie MS (2008) Classifying Variants of Undetermined Significance in BRCA2 with protein likelihood ratios. Cancer Inform 6: 203–16. Epub 2008 Apr 18. 10.4137/cin.s618 PMC2587343 19043619

[58] Li L, Biswas K, Habib LA, Kuznetsov SG, Hamel N, et al (2009) Functional redundancy of exon 12 of BRCA2 revealed by a comprehensive analysis of the c.6853A>G (p.I2285V) variant. Hum Mutat 30: 1543–50. 19795481 10.1002/humu.21101 PMC3501199

[59] Spurdle AB, Healey S, Devereau A, Hogervorst FB, Monteiro AN, et al (2012) ENIGMA–evidence-based network for the interpretation of germline mutant alleles: an international initiative to evaluate risk and clinical significance associated with sequence variation in BRCA1 and BRCA2 genes. Hum Mutat 33: 2–7. 21990146 10.1002/humu.21628 PMC3240687

