# Supplementary material for: Comparative In Vitro and In Silico Analyses of Variants in Splicing Regions of BRCA1 and BRCA2 Genes and Characterization of Novel Pathogenic Mutations
Source: PLoS One. 2013 Feb 22;8(2):e57173. doi: 10.1371/journal.pone.0057173 (PMC3579815; doi:10.1371/journal.pone.0057173)
Supplement: Table S1 — Primers used for mRNA transcripts analysis. (DOCX) [file pone.0057173.s001.docx]

**Supporting information**

**Table S1.** Primers used for mRNA transcript analysis.

| **Variant** | | **Forward PCR Primer** | | **Reverse PCR Primer** | | **Expected amplicon size (bp)** |
| --- | --- | --- | --- | --- | --- | --- |
| **HGVS-nomenclature** | **Location** | **Sequence** | **Location** | **Sequence** | **Location** |  |
| *BRCA1* |  |  |  |  |  |  |
| c.134+3_134+6delAAGT | intron 3 | 5′-tatctgctcttcgcgttg -3′ | exon 2 | 5′-TCTGGTTGAGAAGTTTCAGC-3′ | exon 5 | 156 |
| c.212G>A | exon 5 | 5′-tatctgctcttcgcgttg -3′ | exon 2 | 5′-TCCAAACCTGTGTCAAGCTG-3′ | exon 6 | 292 |
| c.213-11T>G | intron 5 | 5′-ggccttcacagtgtcctt-3′ | exon 5 | 5′-GAGTTGGACACTGAGACTGG-3′ | exon 8 | 299 |
| c.441+2T>G | intron 7 | 5′-aaccaaaaggagcctacaa-3′ | exons 5-6 | 5′-GAGGTTGTATCCGCTGCTT-3′ | exon 8 | 317 |
| c.547+2T>A | intron 8 | 5′-agacttctacagagtgaacc-3′ | exon 7 | 5′-CAATTCTTGATCTCCCAC-3′ | exon 10 | 207 |
| c.548-3delT | intron 8 | 5′-agacttctacagagtgaacc-3′ | exon 7 | 5′-GAGTCATCAGAACCTAACAG -3′ | exon 11 | 788 |
| c.594-4A>G | intron 9 | 5′-gaagataccgttaataaggc-3′ | exon9 | 5′-GAGTCATCAGAACCTAACAG -3′ | exon 11 | 632 |
| c.4097G>A | exon 12 | 5′-gaattggaagacttgactgc-3′ | exon 11 | 5′-TCTGGATTCTGGCTTATAGG-3′ | exon 14 | 506 |
| c.4484G>T | exon 14 | 5′-GGATACCATGCAACATAACC-3′ | exon 13 | 5′-GCCTTGGCAAGTAAGATG-3′ | exon 15 | 476 |
| c.4986+1G>T | intron 16 | 5′-ctcaagaggagctcattaag-3′ | exon 15 | 5′-CAACATGAGTAGTCTCTTCAG-3′ | exon 17 | 492 |
| c.4986+5G>A | intron 16 | 5′-ctcaagaggagctcattaag-3′ | exon 15 | 5′-CAACATGAGTAGTCTCTTCAG-3′ | exon 17 | 492 |
| c.4987-1G>A | intron 16 | 5′-ctcaagaggagctcattaag-3′ | exon 15 | 5′-CTTTAATAGACTGGGTCACC-3′ | exon 19 | 603 |
| c.5278-2delA | intron 20 | 5′-GGGTGACCCAGTCTATTAAAG-3′ | exon 19 | 5′-CACCACAGAAGCACCACAC-3′ | exon 22 | 224 |
| c.5332+1G>A | intron 21 | 5′-GGGTGACCCAGTCTATTAAAG-3′ | exon 19 | 5′-CACCACAGAAGCACCACAC-3′ | exon 22 | 224 |
| c.5333A>G | exon 22 | 5′-gaagtcagaggagatgtgg-3′ | exon 20 | 5′-GGTAGAGTGCTACACTGTCC-3′ | exon 24 | 334 |
| *BRCA2* |  |  |  |  |  |  |
| c.475+1G>A | intron 5 | 5′-GCTGTACCAATCTCCTGTAA-3′ | exon 3 | 5′-CTGACTATGAGCACAGTAGA-3′ | exons 7-8 | 369 |
| c.476-2A>G | intron 5 | 5′-GCTGTACCAATCTCCTGTAA-3′ | exon 3 | 5′-CTGACTATGAGCACAGTAGA-3′ | exons 7-8 | 369 |
| c.631G>A | exon 7 | 5′-GAGATAAGTCAGTGGTATGTGG-3′ | exons 5-6 | 5′-tgtctgtcacagaagcgat-3′ | exon 9 | 294 |
| c.7008-2A>T | intron 13 | 5′-cactccagatggcacaataa-3′ | exons 12-13 | 5′-cctgtatatctctggcattc-3′ | exon 15 | 548 |
| c.8754+3G>C | intron 21 | 5′-tggagaagacatcatctgga-3′ | exon 20 | 5′-TCCAACTGGATCTGAGCTT-3′ | exon 22 | 349 |
| c.8755-1G>A | intron 21 | 5′-tggagaagacatcatctgga-3′ | exon 20 | 5′-CATCTGAAACCGGTAGTTG-3′ | exons 23-24 | 636 |
| c.8954-1_8955delGTTinsAA | intron 22 | 5′-gagcagttaagagccttgaa-3′ | exon 22 | 5′-GGATTCTGGTCGCCACTG-3′ | exon 25 | 624 |
| c.9116C>T | exon 23 | 5′-gagcagttaagagccttgaa-3′ | exon 22 | 5′-GGATTCTGGTCGCCACTG-3′ | exon 25 | 624 |
| c.9117G>A | exon 23 | 5′-gagcagttaagagccttgaa-3′ | exon 22 | 5′-GGATTCTGGTCGCCACTG-3′ | exon 25 | 624 |

Abbreviations: HGVS, Human Genetic Variation Society (<http://www.hgvs.org/mutnomen/>).
