# Supplementary material for: Comparative In Vitro and In Silico Analyses of Variants in Splicing Regions of BRCA1 and BRCA2 Genes and Characterization of Novel Pathogenic Mutations
Source: PLoS One. 2013 Feb 22;8(2):e57173. doi: 10.1371/journal.pone.0057173 (PMC3579815; doi:10.1371/journal.pone.0057173)
Supplement: Table S2 — Nucleotide changes and primers used to assess the allelic expression of normal transcripts in carriers of analyzed variants. (DOCX) [file pone.0057173.s002.docx]

**Supporting information**

**Table S2.** Nucleotide changes and primers used to assess the allelic expression of normal transcripts in carriers of analyzed variants.

| **Variant** | **Nucleotide change** | | **Forward PCR Primer** | | **Reverse PCR Primer** | | **Sequencing primers if different from PCR primers** | |
| --- | --- | --- | --- | --- | --- | --- | --- | --- |
| **HGVS-nomenclature** | **HGVS-nomenclature** | **Location** | **Sequence** | **Location** | **Sequence** | **Location** | **Sequence** | **Location** |
| *BRCA1* |  |  |  |  |  |  |  |  |
| c.212G>A | c.212G>A | exon 5 | 5′-gtgtcctttatgtaagaatg-3′ | exon 5 | 5′-TCCAAACCTGTGTCAAGCTG-3′ | exon 6 |  |  |
| c.213-11T>G | c.2082C>T | exon 11 | 5′-aaccaaaaggagcctacaa-3′ | exons 5-6 | 5′-TGGAAGGCTAGGATTGAC-3′ | exon 11 | 5′-gcgcttgaactagtagtcag-3′ | exon 11 |
| c.441+2T>G | c.2082C>T | exon 11 | 5′-agacttctacagagtgaacc-3′ | exon 7 | 5′-TGGAAGGCTAGGATTGAC-3′ | exon 11 | 5′-gcgcttgaactagtagtcag-3′ | exon 11 |
| c.547+2T>A | c.2082C>T | exon 11 | 5′-CCAGTCTCAGTGTCCAACTC-3′ | exon 8 | 5′-TGGAAGGCTAGGATTGAC-3′ | exon 11 | 5′-gcgcttgaactagtagtcag-3′ | exon 11 |
| c.548-3delT | c.1067A>G | exon 11 | 5′-agacttctacagagtgaacc-3′ | exon 7 | 5′-GAGTCATCAGAACCTAACAG-3′ | exon 11 |  |  |
| c.594-4A>G | c.2082C>T | exon 11 | 5′-gggatgaaatcagtttgg-3′ | exon 10 | 5′-TGGAAGGCTAGGATTGAC-3′ | exon 11 | 5′-gcgcttgaactagtagtcag-3′ | exon 11 |
| c.4097G>A | c.4097G>A | exon 12 | 5′-gaattggaagacttgactgc-3′ | exon 11 | 5′-TCTGGATTCTGGCTTATAGG-3′ | exon 14 |  |  |
| c.4484G>T | c.4484G>T | exon 14 | 5′-CCTATAAGCCAGAATCCAGA-3′ | exon 14 | 5′-GATTCCAGATTCCAGGTAAGG-3′ | exon 16 |  |  |
| c.4986+5G>A^a^ | c.3119G>A | exon 11 | 5′-GTGAACTTGATGCTCAGTAT-3′ | exon 11 | 5′-CAACATGAGTAGTCTCTTCAG-3′ | exon 17 | 5′-CTAGGACTCCTGCTAAGCTC-3′ | exon 11 |
| c.4987-1G>A | c.4837A>G | exon 16 | 5′-gatccttctgaagacagagc-3′ | exon 16 | 5′-CAACATGAGTAGTCTCTTCAG-3′ | exon 17 |  |  |
| c.5278-2delA | c.4837A>G | exon 16 | 5′-gatccttctgaagacagagc-3′ | exon 16 | 5′-ATTTCTAGCCCCCTGAAG-3′ | exon 21 | 5′-GACACCACCATGGACATTC-3′ | exon 16 |
| c.5332+1G>A | c.4837A>G | exon 16 | 5′-cataccatcttcaacctctgc-3′ | exon 16 | 5′-GGCCCATAGCAACAGATTTC-3′ | exon 21 | 5′-CACGAGCATAAATTCTTCTGG-3′ | exon 16-17 |
| c.5333A>G | c.5333A>G | exon 22 | 5′-gaagtcagaggagatgtgg-3′ | exon 20 | 5′-GGTAGAGTGCTACACTGTCC-3′ | exon 24 |  |  |
| *BRCA2* |  |  |  |  |  |  |  |  |
| c.475+1G>A | c.1-26G>A | 5′-UTR | 5′-gccgggagaagcgtgagggg-3′ | 5’-UTR | 5′-CCACATACCACTGACTTATC-3′ | exons 5-6 |  |  |
| c.476-2A>G | c.1114C>A | exon 10 | 5′-GGGAGTTTGTTTCATACACC-3′ | exon 6 | 5′-AGTTGAGACCATTCACAGG-3′ | exon 10 | 5′-gtaaatagctgcaaagacca-3′ | exon 10 |
| c.631G>A | c.631G>A | exon 7 | 5′-AAGTCTAGGAGCTGAGGTGG-3′ | exon 7 | 5′-tgtctgtcacagaagcgat-3′ | exon 9 |  |  |
| c.7008-2A>T | c.7242A>G | exon 14 | 5′-tgtaccctttcgcacaacta-3′ | exons13-14 | 5′-GCTTTTGTCTGTTTTCCTCCAA-3′ | exon 14 |  |  |
| c.8754+3G>C | c.1114C>A | exon 10 | 5′-TGTATCTGAAGTGGAACCAA-3′ | exon 10 | 5′-cactgaaataaccctcaagg-3′ | exons 21-22 | 5′-AGTTGAGACCATTCACAGG-3′ | exon 10 |
| c.8755-1G>A | c.9876G>A | exon 27 | 5′-gagcagttaagagccttgaa-3′ | exon 22 | 5′-ATGGAGTCATCTGAGGAGAA-3′ | exon 27 | 5′-TTCCACACCTGTCTCAGC-3′ | exon 27 |
| c.8954-1_8955delGTTinsAA | c.10362A>C | 3′-UTR | 5′-TTGGCGTCCATCATCAGAT-3′ | exon 23 | 5′-gTATACCAATACGGAATCGG-3′ | exon 27 | 5′-CTgtcagtgaatccactagg-3′ | exon 10 |
| c.9116C>T | c.9116C>T | exon 23 | 5′-gagcagttaagagccttgaa-3′ | exon 22 | 5′-GGATTCTGGTCGCCACTG-3′ | exon 25 |  |  |
| c.9117G>A | c.9117G>A | exon 23 | 5′-TTGGCGTCCATCATCAGAT-3′ | exon 23 | 5′-GGATTCTGGTCGCCACTG-3′ | exon 25 |  |  |

^a^ For this variant, it was not possible to design a reverse primer that annealed to a sequence exclusive of the normal cDNA. The amplification products obtained with the indicated primers were cloned into plasmid vectors and the occurrence of mono- or bi-allelic expression was verified inspecting the sequences of clones containing the normal transcript. Abbreviations: HGVS, Human Genetic Variation Society (<http://www.hgvs.org/mutnomen/>); UTR, untranslated region.
