# Supplementary material for: Comparative In Vitro and In Silico Analyses of Variants in Splicing Regions of BRCA1 and BRCA2 Genes and Characterization of Novel Pathogenic Mutations
Source: PLoS One. 2013 Feb 22;8(2):e57173. doi: 10.1371/journal.pone.0057173 (PMC3579815; doi:10.1371/journal.pone.0057173)
Supplement: Table S3 — In silico analyses of group B variants. (DOCX) [file pone.0057173.s003.docx]

**Supporting information**

**Table S3.** *In silico* analyses of group B variants.

| **Variant**  **HGVS-nomenclature** | **SSF** | |  | **MES** | |  | **NNSPLICE** | |  | **GS** | |  | **HSF** | |  | **NG2** | |  | **SV** | |  | **SP** | |  | **ASSA** | |
| --- | --- | --- | --- | --- | --- | --- | --- | --- | --- | --- | --- | --- | --- | --- | --- | --- | --- | --- | --- | --- | --- | --- | --- | --- | --- | --- |
|  | **wt SSPS** | **variant SSPS** |  | **wt SSPS** | **variant SSPS** |  | **wt SSPS** | **variant SSPS** |  | **wt SSPS** | **variant SSPS** |  | **wt SSPS** | **variant SSPS** |  | **wt SSPS** | **variant SSPS** |  | **wt SSPS** | **variant SSPS** |  | **wt SSPS** | **variant SSPS** |  | **wt**  **Ri** | **variant Ri** |
| *BRCA1* |  |  |  |  |  |  |  |  |  |  |  |  |  |  |  |  |  |  |  |  |  |  |  |  |  |  |
| c.134+3_134+6del AAGT | 87.86 | nr |  | 10.08 | nr |  | 0.89 | nr |  | 2.61 | nr |  | 87.28 | 64.90 |  | 0.54 | nr |  | 84. | nr |  | 0.976 | nr |  | 9.1 | 0.5 |
| c.212G>A | 77.37 | nr |  | 7.84 | 1.45 |  | 0.92 | nr |  | 2.81 | nr |  | 78.08 | 67.50 |  | 0.37 | nr |  | 79. | nr |  | 0.860 | nr |  | 6.0 | 2.9 |
| c.213-11T>G | 85.51 | nr |  | 4.84 | nr |  | nr | nr |  | nr | nr |  | 89.39 | 85.73 |  | 0.23 | nr |  | 78. | nr |  | nr | nr |  | 3.8 | 1.4 |
| c.548-3delT | nr | nr |  | 2.82 | 1.70 |  | nr | nr |  | nr | nr |  | 75.71 | 73.98 |  | nr | nr |  | nr | nr |  | 0.960 | 0.966 |  | 0.5 | 0.5 |
| c.594-4A>G | 83.28 | 83.28 |  | 10.00 | 10.15 |  | 0.98 | 0.97 |  | 4.51 | 5.12 |  | 86.87 | 86.80 |  | 0.15 | nr |  | 88. | 88. |  | 0.994 | 0.995 |  | 10.2 | 10.3 |
| c.4097G>A | 89.19 | 85.28 |  | 8.15 | 6.24 |  | 0.89 | 0.76 |  | 10.70 | 9.39 |  | 83.03 | 79.90 |  | nr | nr |  | 84. | 82. |  | 1.000 | 0.999 |  | 8.8 | 7.9 |
| c.4484G>T | 94.36 | 81.75 |  | 10.57 | 5.55 |  | 1.00 | 0.90 |  | 2.15 | nr |  | 96.71 | 85.84 |  | 0.99 | 0.55 |  | 89. | 83. |  | 0.966 | nr |  | 11.0 | 7.7 |
| c.4986+5G>A | 70.38 | nr |  | 5.91 | nr |  | 0.66 | nr |  | nr | nr |  | 81.24 | 69.08 |  | 0.89 | nr |  | 82. | nr |  | nr | nr |  | 5.7 | 2.2 |
| c.5333A>G | 73.01 | 76.92 |  | 8.67 | 9.73 |  | 0.67 | 0.89 |  | 6.38 | 7.51 |  | 80.46 | 83.59 |  | 0.43 | 0.43 |  | 77. | 79. |  | 0.999 | 0.999 |  | 4.3 | 5.2 |
| *BRCA2* |  |  |  |  |  |  |  |  |  |  |  |  |  |  |  |  |  |  |  |  |  |  |  |  |  |  |
| c.631G>A | 78.15 | nr |  | 6.84 | nr |  | 0.44 | nr |  | nr | nr |  | 83.37 | 72.79 |  | nr | nr |  | 81. | nr |  | nr | nr |  | 5.6 | 2.6 |
| c.8754+3G>C | 87.25 | 81.73 |  | 7.66 | 5.24 |  | 0.98 | 0.63 |  | 3.23 | nr |  | 91.88 | 85.06 |  | 1.00 | 0.71 |  | 88. | 81. |  | 0.940 | nr |  | 10.0 | 5.6 |
| c.9116C>T | nr | nr |  | 4.28 | 2.41 |  | 0.57 | nr |  | nr | nr |  | 71.94 | 71.94 |  | nr | nr |  | 75. | 75. |  | 0.897 | nr |  | 2.5 | 2.4 |
| c.9117G>A | nr | nr |  | 4.28 | nr |  | 0.57 | nr |  | nr | nr |  | 71.94 | 61.37 |  | nr | nr |  | 75. | nr |  | 0.897 | nr |  | 2.5 | -0.5 |
| Non Informative Analyses (%) | 23.1 (3/13) | |  | 0.0 (0/13) | |  | 15.4 (2/13) | |  | 46.1 (6/13) | |  | 0.0 (0/13) | |  | 38.5 (5/13) | |  | 7.7 (1/13) | |  | 23.1 (3/13) | |  | 0.0 (0/13) | |

For each computational program, SSPS/Ri values of natural splice sites in the wild-type (wt) and variant sequences are reported. Abbreviations: HGVS, Human Genetic Variation Society (http://www.hgvs.org/mutnomen/); SSPS, splice site prediction score; Ri, information value; nr, not recognized.
