# Supplementary material for: Comparative In Vitro and In Silico Analyses of Variants in Splicing Regions of BRCA1 and BRCA2 Genes and Characterization of Novel Pathogenic Mutations
Source: PLoS One. 2013 Feb 22;8(2):e57173. doi: 10.1371/journal.pone.0057173 (PMC3579815; doi:10.1371/journal.pone.0057173)
Supplement: Table S4 — In silico analyses of spliceogenic variants leading to the activation/creation of alternative splice sites. (DOCX) [file pone.0057173.s004.docx]

**Supporting information**

**Table S4.** *In silico* analyses of spliceogenic variants leading to the activation/creation of alternative splice sites.

| **Variant**  **HGVS-nomenclature** | **Location of natural and alternative SSs** | **SSF** | |  | **MES** | |  | **NNSPLICE** | |  | **GS** | |  | **HSF** | |  | **NG2** | |  | **SV** | |  | **SP** | |  | **ASSA** | |
| --- | --- | --- | --- | --- | --- | --- | --- | --- | --- | --- | --- | --- | --- | --- | --- | --- | --- | --- | --- | --- | --- | --- | --- | --- | --- | --- | --- |
|  |  | **wt SSPS** | **variant SSPS** |  | **wt SSPS** | **variant SSPS** |  | **wt SSPS** | **variant SSPS** |  | **wt SSPS** | **variant SSPS** |  | **wt SSPS** | **variant SSPS** |  | **wt SSPS** | **variant SSPS** |  | **wt SSPS** | **variant SSPS** |  | **wt SSPS** | **variant SSPS** |  | **wt**  **Ri** | **variant Ri** |
| *BRCA1* |  |  |  |  |  |  |  |  |  |  |  |  |  |  |  |  |  |  |  |  |  |  |  |  |  |  |  |
| c.212G>A | *c.212* | 77.37 | nr |  | 7.84 | 1.45 |  | 0.92 | nr |  | 2.81 | nr |  | 78.08 | 67.50 |  | 0.37 | nr |  | 79. | nr |  | 0.860 | nr |  | 6.0 | 2.9 |
|  | **c.190** | 77.93 | 77.93 |  | 3.54 | 3.54 |  | 0.91 | 0.91 |  | nr | nr |  | 84.81 | 84.81 |  | 0.32 | 0.37 |  | 79. | 79. |  | nr | nr |  | 6.2 | 6.2 |
| c.213-11T>G | *c.213* | 85.51 | nr |  | 4.84 | nr |  | nr | nr |  | nr | nr |  | 89.39 | 85.73 |  | 0.23 | nr |  | 78. | nr |  | nr | nr |  | 3.8 | 1.4 |
|  | **c.213-59** | 86.74 | 86.74 |  | 10.23 | 10.23 |  |  | |  |  | |  | 82.22 | 82.22 |  | 0.27 | 0.27 |  | 85. | 85. |  |  | |  | 9.2 | 9.2 |
| c.441+2T>G | *c.441* | nr | nr |  | 3.23 | nr |  | nr | nr |  | nr | nr |  | 78.77 | nr |  | nr | nr |  | 76. | nr |  | 0.923 | nr |  | 3.7 | -4.5 |
|  | **c.379** |  | |  | 3.77 | 3.77 |  |  | |  |  | |  | 77.73 | 77.73 |  |  | |  | nr | nr |  | nr | nr |  | 5.6 | 5.6 |
| c.4986+1G>T | *c.4986* | 70.38 | nr |  | 5.91 | nr |  | 0.66 | nr |  | nr | nr |  | 81.24 | nr |  | 0.89 | nr |  | 82. | nr |  | nr | nr |  | 5.7 | -2.1 |
|  | **c.4986+65** | 73.18 | 73.18 |  | 2.16 | 2.16 |  | nr | nr |  |  | |  | 78.81 | 78.81 |  | nr | nr |  | 79. | 79. |  |  | |  | 5.3 | 5.3 |
| c.4986+5G>A | *c.4986* | 70.38 | nr |  | 5.91 | nr |  | 0.66 | nr |  | nr | nr |  | 81.24 | 69.08 |  | 0.89 | nr |  | 82. | nr |  | nr | nr |  | 5.7 | 2.2 |
|  | **c.4986+65** | 73.18 | 73.18 |  | 2.16 | 2.16 |  | nr | nr |  |  | |  | 78.81 | 78.81 |  | nr | nr |  | 79. | 79. |  |  | |  | 5.3 | 5.3 |
| c.5278-2delA | *c.5278* | 90.05 | nr |  | 13.07 | nr |  | 0.99 | nr |  | 14.41 | nr |  | 93.64 | nr |  | 1.00 | nr |  | 96. | nr |  | 0.999 | nr |  | 17.9 | 10.5 |
|  | **c.5286** | nr | 87.21 |  | 1.96 | 9.23 |  | 0.85 | 0.96 |  | nr | 7.64 |  | 86.32 | 90.06 |  | 0.81 | 0.85 |  | 87. | 91. |  | nr | 0.978 |  | 11.9 | 14.6 |
| *BRCA2* |  |  |  |  |  |  |  |  |  |  |  |  |  |  |  |  |  |  |  |  |  |  |  |  |  |  |  |
| c.8754+3G>C | *c.8754* | 87.25 | 81.73 |  | 7.66 | 5.24 |  | 0.98 | 0.63 |  | 3.23 | nr |  | 91.88 | 85.06 |  | 1.00 | 0.71 |  | 88. | 81. |  | 0.940 | nr |  | 10.0 | 5.6 |
|  | **c.8754+46** | 90.00 | 90.00 |  | 8.68 | 8.68 |  | 1.00 | 1.00 |  | 6.71 | 6.64 |  | 95.55 | 95.55 |  | 0.63 | 0.79 |  | 89. | 89. |  | 0.991 | 0.991 |  | 10.6 | 10.6 |
| c.8755-1G>A | *c.8755* | 77.58 | nr |  | 3.78 | nr |  | nr | nr |  | 1.75 | nr |  | 81.68 | nr |  | 0.33 | nr |  | nr | nr |  | 0.931 | nr |  | 3.3 | -4.3 |
|  | **c.9005** | 88.08 | 88.08 |  | 7.86 | 7.86 |  |  | |  | nr | nr |  | 86.41 | 86.41 |  | 0.41 | 0.41 |  |  | |  | 0.939 | 0.939 |  | 7.5 | 7.5 |
| c.8954-1_8955 delGTTinsAA | *c.8954* | 82.36 | nr |  | 10.35 | nr |  | 0.53 | nr |  | 3.75 | nr |  | 87.47 | nr |  | nr | nr |  | 84. | nr |  | 0.987 | nr |  | 10.1 | 3.3 |
|  | **c.9005** | 88.08 | 88.08 |  | 7.86 | 7.86 |  | 0.86 | 0.86 |  | nr | nr |  | 86.41 | 86.41 |  |  | |  | 84. | 84. |  | 0.939 | 0.939 |  | 7.5 | 7.5 |
| c.7008-2A>T | *c.7008* | 82.03 | nr |  | 10.37 | nr |  | 0.56 | nr |  | 4.45 | nr |  | 87.48 | nr |  | 0.31 | nr |  | 87. | nr |  | 0.995 | nr |  | 11.8 | 4.4 |
|  | **c.7018** | nr | nr |  | nr | 0.45 |  | nr | nr |  | nr | nr |  | 71.36 | 74.71 |  | nr | nr |  | nr | nr |  | nr | nr |  | -1.0 | 1.6 |
|  | **c.7254** | 83.54 | 83.54 |  | 4.44 | 4.44 |  | nr | nr |  | nr | nr |  | 84.75 | 84.75 |  | 0.17 | 0.17 |  | 79. | 79. |  | 0.902 | 0.902 |  | 5.1 | 5.1 |

For each computational program, SSPS/Ri values of the natural splice site (in italic) affected by the indicated mutations and of the alternatively used splice sites (in bold) in the wild-type (wt) and variant sequences are reported. The SSPS and Ri values of alternative splice sites were not reported in cases in which the program did not recognize the natural splice site. Abbreviations: HGVS, Human Genetic Variation Society (<http://www.hgvs.org/mutnomen/>); SS, Splice Site; SSPS, splice site prediction score; Ri, information value; nr: not recognized.
