# Supplementary material for: Comparative In Vitro and In Silico Analyses of Variants in Splicing Regions of BRCA1 and BRCA2 Genes and Characterization of Novel Pathogenic Mutations
Source: PLoS One. 2013 Feb 22;8(2):e57173. doi: 10.1371/journal.pone.0057173 (PMC3579815; doi:10.1371/journal.pone.0057173)
Supplement: Table S5 — Location of putative splice sites and corresponding SSPS/Ri values identified by three bioinformatics programs in the BRCA1 and BRCA2 gene regions spanning the natural splice sites affected by the indicated spliceogenic mutations and adjacent sequences. (DOCX) [file pone.0057173.s005.docx]

**Supporting information**

**Table S5.** Location of putative splice sites and corresponding SSPS/Ri values identified by three bioinformatics programs in the *BRCA1* and *BRCA2* gene regions spanning the natural splice sites affected by the indicated spliceogenic mutations and adjacent sequences.

| **Variant**  **HGVS-nomenclature** | **Location of natural and alternative SS(s)** | **Variant sequence SSPSs** | | **Variant sequence Ri** |
| --- | --- | --- | --- | --- |
|  |  | **MES** | **HSF** | **ASSA** |
| ***BRCA1*** |  |  |  |  |
| c.212G>A | c.135-386 |  | 90.40^ |  |
|  | c.135-226 | 8.31^ |  | 8.9^ |
|  | **c.190** | 3.54 | 84.81 | 6.2 |
|  | *c.212* | 1.45 | 67.50 | 2.9 |
|  | c.212+12 | 4.83* | 79.02* | 3.9* |
| c.213-11T>G | **c.213-59** | 10.23^ | 82.22 | 9.2 |
|  | c.213-47 |  |  | 6.0* |
|  | c.213-10 | 1.40* |  |  |
|  | *c.213* | nr | 85.73 | 1.4 |
|  | c.216 |  | 72.38* |  |
|  | c.283 |  | 87.35^ |  |
|  | c.301+282 |  |  | 10.0^ |
| c.441+2T>G | c.302-125 | 8.66^ |  |  |
|  | **c.379** | 3.77* | 77.73 | 5.6 |
|  | c.418 |  | 68.11* | 1.7* |
|  | *c.441* | nr | nr | -4.5 |
|  | c.441+275 |  |  | 8.0^ |
|  | c.441+464 |  | 92.09^ |  |
| c.4986+1G>T | *c.4986* | nr | nr | -2.1 |
|  | c.4986+4 |  | 60.46* |  |
|  | c.4986+16 |  |  | 0.6* |
|  | **c.4986+65** | 2.16* | 78.81 | 5.3 |
|  | c.4986+301 | 7.33^ |  | 7.1^ |
|  | c.4986+451 |  | 82.05^ |  |
| c.4986+5G>A | *c.4986* | nr | 69.08 | 2.2 |
|  | c.4986+6 | 1.97* | 65.19* |  |
|  | c.4986+16 |  |  | 0.6* |
|  | **c.4986+65** | 2.16 | 78.81 | 5.3 |
|  | c.4986+301 | 7.33^ |  | 7.1^ |
|  | c.4986+451 |  | 82.05^ |  |
| c.5278-2delA | *c.5278* | nr | nr | 10.5 |
|  | c.5280 |  |  | 2.5* |
|  | **c.5286** | 9.23*^ | 90.06*^ | 14.6^ |
| ***BRCA2*** |  |  |  |  |
| c.8754+3G>C | *c.8754* | 5.24 | 85.06 | 5.6 |
|  | c.8754+8 | 5.73* | 81.28* | 6.0* |
|  | **c.8754+46** | 8.68^ | 95.55^ | 10.6^ |
| c.8755-1G>A | c.8755-382 |  | 87.82^ |  |
|  | c.8755-18 |  |  | 2.8* |
|  | *c.8755* | nr | nr | -4.3 |
|  | c.8756 | 1.60* | 73.73* |  |
|  | c.8776 |  |  | 10.1^ |
|  | c.8954 | 10.35^ |  |  |
|  | **c.9005** | 7.86 | 86.41 | 7.5 |
| c.8954-1_8955delGTTinsAA | c.8954-3 |  |  | 2.4* |
|  | *c.8954* | nr | nr | 3.3 |
|  | c.8964 |  | 65.06* |  |
|  | c.8984 | 4.61* |  |  |
|  | **c.9005** | 7.86 | 86.41 | 7.5 |
|  | c.9118 | 12.19^ |  | 13.9^ |
|  | c.9142 |  | 89.02^ |  |
| c.7008-2A>T | c.7008-3 |  |  | 2.5* |
|  | *c.7008* | nr | nr | 4.4 |
|  | c.7011 |  |  | 3.5* |
|  | **c.7018** | 0.45* | 74.71* | 1.6 |
|  | **c.7254** | 4.44 | 84.75 | 5.1 |
|  | c.7435+42 | 7.38^ | 91.29^ | 7.7^ |

For the three indicated programs, the SSPS/Ri values in the mutated sequences of the natural splice sites (in italic) affected by the indicated mutations, those of alternatively used splice sites (in bold) and of other putative splice sites within the gene sequences spanning ±500 bp from the natural splice sites were considered. The putative splice site closest to the abrogated natural splice site (*) or with the highest prediction scores (^) are indicated. Abbreviations: HGVS, Human Genetic Variation Society (<http://www.hgvs.org/mutnomen/>); SS, Splice Site; SSPS, splice site prediction score; Ri, information value; nr, not recognized.
