# Supplementary material for: Comparative In Vitro and In Silico Analyses of Variants in Splicing Regions of BRCA1 and BRCA2 Genes and Characterization of Novel Pathogenic Mutations
Source: PLoS One. 2013 Feb 22;8(2):e57173. doi: 10.1371/journal.pone.0057173 (PMC3579815; doi:10.1371/journal.pone.0057173)
Supplement: Table S6 — Spliceogenic variants for which different transcripts patterns were observed in the present compared with previous studies and experimental details. (DOCX) [file pone.0057173.s006.docx]

**Supporting information**

**Table S6.** Spliceogenic variants for which different transcript patterns were observed in the present compared with previous studies and experimental details.

| **Variant**  **HGVS-nomenclature** | **mRNA change observed** | **mRNA method** | **mRNA source** | **Primer Location**  **Forward Reverse** | | **Reference** |
| --- | --- | --- | --- | --- | --- | --- |
| *BRCA2* |  |  |  |  |  |  |
| c.631G>A | skipping of exon 7 | RT-PCR | LCLs with puromycin | exons 5-6 | exon 9 | present study |
|  | skipping of exon 7; skipping of 70 bp at 5′-end of exon 7 | minigene | ns | intron 6 | intron 7 | [19] |
| c.7008-2A>T | skipping of exon 14; skipping of 10 bp at 5′-end of exon 14; skipping of 246 bp at 5′-end of exon 14 | RT-PCR | LCLs with puromycin | exons 12-13 | exon 15 | present study |
|  | skipping of 10 bp at 5′-end of exon 14 | RT-PCR | whole blood | ns | ns | [22] |
|  | skipping of exon 14 | RT-PCR | PBLs | exon 13 | exon 15 | [45] |
| c.8755-1G>A | skipping of exon 22 + 51 bp at 5′-end of exon 23 | RT-PCR | LCLs with puromycin | exon 20 | exon 23-24 | present study |
|  | skipping of exon 22 | RT-PCR | short-term PHA-stimulated lymphocyte cultures | ns | ns | [43] |
| c.8954-1_8955delGTTinsAA | skipping of 51 bp at 5′-end of exon 23; skipping of exon 23 | RT-PCR | LCLs with puromycin | exon 22 | exon 25 | present study |
|  | skipping of 51 bp at 5′-end of exon 23; skipping of exon 23; skipping of exons 23-24 | minigene | ns | intron 18 | intron 20 | [18] |
| c.9117G>A | skipping of exon 23 | RT-PCR | LCLs with puromycin | exon 22 | exon 25 | present study |
|  | skipping of exon 23; skipping of 51 bp at 5′-end of exon 23; skipping of exons 23-24 | minigene | ns | intron 18 | intron 20 | [18] |

Abbreviations: PBL, peripheral blood lymphocytes; PHA, phytohaemagglutinin; ns, not specified.
